# Correlates of physical activity in adults with spondyloarthritis and rheumatoid arthritis: a systematic review

**DOI:** 10.1007/s00296-022-05142-z

**Published:** 2022-06-08

**Authors:** Thomas Ingram, Raj Sengupta, Martyn Standage, Rosie Barnett, Peter Rouse

**Affiliations:** 1 Department for Health, University of Bath, Bath, UK; 2 Royal National Hospital for Rheumatic Diseases, Royal United Hospitals NHS Foundation Trust, Bath, UK; 3 Department of Pharmacy & Pharmacology, University of Bath, Bath, UK

**Keywords:** Physical activity, Correlate, Factor, Rheumatoid arthritis, Spondyloarthritis

## Abstract

Physical activity (PA) is a primary non-pharmacological treatment option for those living with rheumatoid arthritis (RA) and spondyloarthritis (SpA). The aim of this systematic literature review was to summarize and present an updated synthesis of the factors associated with PA in the RA and SpA populations. A tailored search of PubMed (inc. Medline), Web of Science, Embase, APA PsycNET, and Scopus was conducted for research published between 2004 and June 2019. Methodological quality was assessed using The National Institutes of Health (NIH) Quality Assessment Tools for Observational Cohort and Cross-sectional Studies, Case–Control Studies, and Controlled Intervention Studies. Forty RA and eleven SpA articles met the inclusion criteria. Methodological quality was generally fair to good, with two RA studies rated as poor. Correlates are discussed in the sociodemographic, physical, psychological, social, and environmental categories. Environmental factors were not measured in any RA study. In individuals living with RA, consistent positive associations were found between PA and high-density lipoprotein, self-efficacy, and motivation. Consistent negative associations were found for functional disability and fatigue. In individuals with SpA, consistent positive associations were found between PA and quality of life, and consistent negative associations with functional disability. Physical and psychological factors are most consistently related with PA parameters in those living with RA and SpA. Many variables were inconsistently studied and showed indeterminant associations. Studies with prospective designs are needed to further understand the factors associated with PA in these populations, especially in those living with SpA.

**Supplementary Information:**

The online version contains supplementary material available at 10.1007/s00296-022-05142-z.

## Introduction

Spondyloarthritis (SpA) and rheumatoid arthritis (RA) are chronic inflammatory diseases which share the occurrence of fluctuating flares and stable disease activity [1–4]. Clinically, those with SpA (specifically axial spondyloarthritis) often suffer from inflammatory back pain, sacroiliitis, stiffness, and extreme fatigue [5], whereas those with RA often suffer from joint swelling, cartilage damage and synovial joint destruction [6]. Both inflammatory conditions cause disability and are associated with work challenges [7, 8] and reduced health-related quality of life [9]. The prevalence of SpA ranges from 0.20% in South-East Asia to 1.61% in Northern Arctic communities, whereas the prevalence of ankylosing spondylitis (AS) ranges from 0.02% in Sub-Saharan Africa to 0.35% in Northern Arctic communities [10]. In terms of RA, results of a meta-analyses of 67 studies showed the global prevalence to be 460 per 100,000 between the years 1980 to 2019 [11].

Progressive and beneficial pharmacological treatments for SpA and RA include non-steroidal anti-inflammatories, biologic and targeted synthetic disease-modifying antirheumatic drugs [12, 13]. However, in recent European League Against Rheumatism (EULAR) recommendations, physical activity (PA) has been proposed as an integral option for the non-pharmacological management of inflammatory arthritides [14]. The latest World Health Organizations (WHO) 2020 guidelines on physical activity and sedentary behaviour [15] are the same for healthy adults and those with chronic conditions. Here, the recommendations are that all adults should undertake at least 150 to 300 min of moderately intense aerobic PA or 75 to 150 min of vigorously intense PA, whilst also conducting muscle-strengthening PA at least 2 days per week on all major muscle groups. For adults aged 65 years and older, multicomponent PA focusing on strength training and functional balance is also recommended. Despite the recommendations, those with SpA and RA have been shown to be more sedentary and less physically active than their healthy counterparts [16, 17].

People living with SpA and RA receive similar benefits to their healthy counterparts from engaging in regular PA, including increased muscle strength and cardiovascular fitness [18]. However, supplementary benefits of PA in the SpA and RA population include a reduction in disease activity and inflammation [19, 20]. While the aforementioned studies demonstrate how PA interventions can improve general and disease-related health of individuals with RA and SpA, challenges arise both before and after the PA intervention itself. Adherence to PA after intervention is an example of one challenge in the SpA and RA populations [21, 22], yet this review will focus on the initial challenge of identifying the factors that associate with PA behaviour. Correlates of PA provide clinically relevant information that needs early consideration in intervention design to maximise the potential benefits of a PA intervention.

Despite a growing body of research on the correlates of PA, little research has collated the data in those with SpA. Wilcox et al. [23] initially evaluated the correlates of PA in people living with inflammatory arthritis, however, only two studies were identified that included SpA participants. In narrowing the focus to just people living with RA, a more recent review by Larkin and Kennedy [24] reported positive associations between PA and health perception, self-efficacy, motivation, and levels of previous PA. In addition, coerced regulation style, fatigue, and specific physiological variables were negatively associated with PA. Nevertheless, Larkin and Kennedy [24] concluded there were no definite correlates of PA, prompting the need for more research to aid clinical practitioners in supporting PA behaviour in those with RA. To address the current lack of synthesis of PA correlates in those with SpA and the advancing research utilizing objective measures of PA in inflammatory rheumatic conditions, an updated comprehensive review is needed to allow a better understanding of the factors related to PA in people living with SpA and RA. Therefore, the aim of the present work is to provide an updated synthesis of the factors most strongly associated with PA among people living with SpA and RA.

## Methods

The protocol for this review was registered online with a prospective registry of systematic reviews (PROSPERO ID: CRD42019138993). Where possible, the ‘Preferred Reporting Items for Systematic Reviews and Meta-analyses’ (PRISMA) recommendations [25] were followed.

### Search strategy

A systematic search was conducted using a tailored search strategy in the following databases: PubMed (inc. Medline), Web of Science, Embase, APA PsycNET, and Scopus. The search strategy was developed based on a preliminary search in PubMed to identify relevant terminology, search terms employed by similar reviews into correlates of PA in inflammatory arthritis, and discussions with a subject librarian and the research team. The databases were searched from 2004 to June 2019, to develop and further a review previously conducted by Larkin and Kennedy [24]. An example of the search strategy used is shown below. The search was restricted to articles with human participants and published in English. Additional eligible studies the author became aware of were included.

#### Example search strategy for PubMed (inc. Medline)

(Spondyloarthr* OR spondyloarthritis[mh] OR “ankylosing spondylitis*” OR ankylosing spondylitis[mh] OR “rheumatoid arthritis” OR rheumatoid arthritis[mh]) AND (“physical activity” OR physical activity[mh] OR exercis* OR exercise[mh] OR stretch* OR yoga* OR train*) AND (determinant* OR correl* OR factor* OR predict* OR participat* OR “exercise belief”) All others are [all fields].

*indicates the use of all possible suffixes.

### Selection criteria

Quantitative observational studies and experimental studies that explored correlates of PA in adults (≥ 18 years) with SpA and/or RA were included. Intervention studies were eligible for inclusion if baseline data were reported. Study participants must have had a diagnosis of RA using the American College of Rheumatology (ACR) /EULAR criteria [26]. Due to the limited number of studies, those with SpA were included if they received a doctor diagnosis, but not if the diagnosis was self-reported. SpA diagnoses that were eligible included: axial spondyloarthritis (axSpA); non-radiographic spondyloarthritis (nr-axSpA); ankylosing spondylitis (AS); psoriatic arthritis (PsA); enteropathic arthritis (EnA); reactive arthritis (ReA); and undifferentiated spondyloarthritis (uSpA). Studies utilising self-reported (questionnaires) and objective measures (accelerometers, pedometers, calorimetry, doubly labelled water, and heart rate monitors) of PA were included. For consistency with other reviews [24], PA was defined in this work as ‘*any bodily movement produced by skeletal muscles that results in energy expenditure*’ [27]. Exercise is a subcategory of PA, that is distinct in definition by its ‘*planned, structured and repetitive*’ nature, with an ‘*objective to improve or maintain physical fitness component(s)*’ [27]. Cross-sectional studies where PA was not the dependent variable were included.

Articles were excluded if they were not published between 2004 and June 2019 and if the study participants were aged younger than 18 years. Qualitative studies and studies that exclusively explored functional or physiological variables of PA in SpA and RA were excluded. Studies that included multiple forms of arthritis but did not analyse the data separately were excluded. Studies that reported on physical inactivity were included, but those reporting exclusively on sedentary behaviour were excluded as sedentary behaviour is independent from physical (in)activity [28]. Longitudinal studies in which PA was not the dependent variable were excluded, unless baseline data were reported. Abstracts, conference proceedings, and grey literature were excluded.

### Screening and data extraction

The first author conducted the database searches and imported them to the reference manager software Endnote, whereby duplicates were removed. The abstracts were then imported into the review management software Covidence [29] and further duplicates removed. The first author screened all titles and abstracts independently against the selection criteria, with a second author independently screening 40% to determine eligibility. The same method was used for the review and selection of full text articles. During these two stages a consensus approach was used to resolve any disputes. All disputes were resolved without the need for consultation with a third author. Data extraction was conducted by the first author. Data such as participant characteristics, study design, measure of PA, variables analysed, statistical test, and statistical output were extracted.

### Methodological quality

Selected articles fulfilling the inclusion criteria were assessed for methodological quality using The National Institutes of Health (NIH) Quality Assessment Tools for Observational Cohort and Cross-sectional Studies and Case–Control studies [30]. These assessment tools are recommended and deemed acceptable [31]. The assessment tools appraise studies based on the following concepts: objectives; population and sample size; timeframe; outcome measure; exposure variables and measures; attrition; blinding; and analysis. Each of the questions were scored as yes, no, cannot determine, not applicable, or not reported as appropriate. A final quality rating of good, fair, or poor is afforded to each study by the reviewer. The lead author independently assessed the quality of studies, with a following two authors independently assessing 50% each. Any disputes were resolved via a consensus method between the authors, and further consultation from a third author was not warranted.

### Data synthesis and coding

Following discussions with the research team, meta-analysis was not conducted due to the nature of included studies (e.g., type of PA and statistics reported). Rather, the coding framework employed by Sallis et al. [32] was used to pool and classify the associations between PA parameters and potential correlates into largely positive/negative ( ±), indeterminant / inconsistent (?) or no association (0). Following this framework, univariate tests were reported, even if multivariate tests were presented. The results will be presented using this approach for consistency across studies. Yet, an associations table utilizing the most adjusted or final statistical model for each variable can be seen in Online Resources 1 and 2. Conceptually similar variables were grouped together, for example, “high-density lipoprotein” (HDL) included, HDL, HDL particle, small particle, and large particle concentration. To be consistent with previous reviews [24], the correlates identified were categorised into the following domains: sociodemographic, physical, psychological, social, and environmental.

For studies utilizing multiple measures of PA (i.e., subjective, objective, or multiple subjective), correlations from all PA measures were included. Both categorical (e.g., active vs. inactive, meeting PA recommendations, health-enhancing PA, exercising ≥ 2/week) and continuous PA parameters (e.g., total PA min, step count, time spent in PA intensities, metabolic equivalent of task, physical activity energy expenditure (PAEE), accelerometer counts) were eligible for inclusion. Continuous parameters of PA are reported above categorical parameters of PA if the same study reports both. However, if a variable was analysed as a potential correlate of a categorical PA parameter, but not of a continuous PA parameter, this was included. Up to two PA parameters were included for each study (indicators of total PA considered first), apart from if multiple measures of PA were used, so that one study population is not overrepresented. If more than two types of PA intensity were reported in a study (i.e., light PA (LPA), moderate-intensity PA (MI-PA), vigorous-intensity PA (VI-PA)), the two most intense categories were included. For accelerometer data, PAEE was considered over total energy expenditure (TEE).

## Results

### Study selection, characteristics and quality

**Fig. 1:**
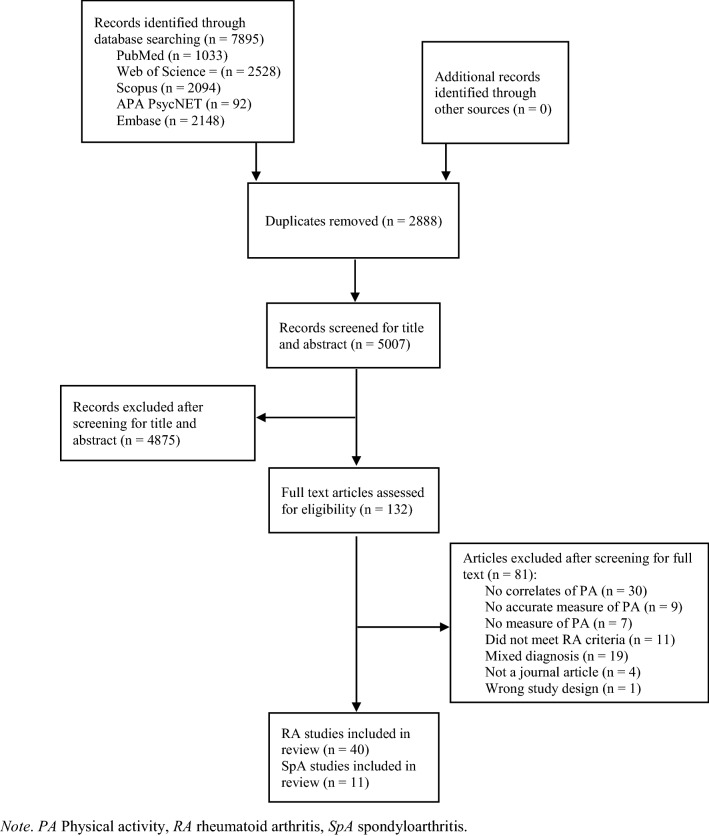
PRISMA flowchart of study screening and selection process

Fifty-one published articles (RA = 40; SpA = 11) were included with the full screening process provided in Fig. 1. The predominant reasons for exclusion were that the studies did not report correlates of PA (*n* = 30) and that the study included mixed diagnoses, non-specific arthritides or did not analyse RA and SpA data separately (n = 19). A description of each study and the overall methodological quality can be seen in Tables 1 and 2. The number of RA participants ranged from 20 to 3,152, whereas the number of SpA participants ranged from 24 to 2,167. Three RA studies utilized participants from the Swedish Rheumatology Quality Registers [35–37], and a further two used participants from the Swedish RA register [40, 41] . Two RA studies contained the same patients from 3 hospitals in The Netherlands [49, 53]. Two other RA studies used participants and cross-sectional data from the same randomized clinical trial investigating the effectiveness of a PA promotion intervention in the USA [38, 54]. One randomized intervention study [63] investigated the effects of a 12-week low-impact aerobic exercise programme on disease and psychological variables, and aerobic fitness, in those with RA. The randomized intervention study compared a class exercise treatment group, a home exercise treatment group (via videotape), and a control group [63]. Two SpA studies included patients from the Skåne Healthcare Register [78, 79]. Most RA studies had a larger proportion of female participants than male, and four RA studies included only female participants [39, 64, 65, 67]. Twelve RA studies were conducted in the United States and twenty-five were conducted in Europe. Ten of the eleven SpA studies were conducted in Europe. The pooled associations and correlates of PA in those with RA and SpA are displayed in Tables 3 and 4. The results are coded in terms of the specific variable and not necessarily the statistical direction derived from the measure of the variable. Online resources 3 and 4 show the positive and negative statistics for each variable related to PA in all included studies.

**Table 1:** Description of included RA studies

Citations	Design	Participants (*n*)	Age (years)	Sex (F)	PA measure	Location	Methodological Quality
Byram et al. [33]	Cross-sectional	RA (165)	54	69%	Customized interview / 2011 Compendium of PA	USA	Fair
Conigliaro et al. [34]	Cohort / case–control	RA (30)	56.07 ± 11.7	25	IPAQ	Italy	Poor
Demmelmaier et al. [35]	Longitudinal	RA (2752)	60 ± 11	2,003	IPAQ-SF (plus ESAI)	Sweden	Good
Demmelmaier et al. [36]	Cross-sectional	RA (3152)	62 (IQR 54–68)	2,309	IPAQ-SF (plus ESAI)	Sweden	Good
Demmelmaier et al. [37]	Longitudinal	RA (2569)	60 ± 11	1,875	IPAQ-SF (plus ESAI)	Sweden	Good
Ehrlich-Jones et al. [38]	Cross-sectional	RA (185)	55 ± 14	155	GT1M Actigraph	USA	Good
Elkan et al. [39]	Cross-sectional	RA (61)	60.8 (57.3–64.4)	61	IPAQ	Sweden	Fair
Eurenius et al. [40]	Cross-sectional	RA (298)	57 (range 19–90)	225	Customized self-report	Sweden	Good
Eurenius et al. [41]	Longitudinal	RA (102)	57 (range 19–84)	76	Customized self-report	Sweden	Good
Fenton et al. [42]	Cross-sectional	RA (61)	54.92 ± 12.39	67.2.%	GT3X Accelerometer—LPA	UK	Good
Greene et al. [43]	Cross-sectional	RA (52)	61 ± 14.5	47	PADS	USA	Good
Hashimoto et al. [44]	Cross-sectional / case control	RA (20)	69.4 ± 5.1	16	Actigraph mini-motionlogger, omnidirectional accelerometer	Japan	Good
Henchoz et al. [45]	Case control / cross-sectional	RA (110)	53% (40–59) 47% (60–80)	83	PAFQ	Switzerland	Good
Hernández-Hernández et al. [46]	Case-control / cross-sectional / longitudinal	RA (50)	54.5 ± 7.4	44	IPAQ and RT3 triaxial accelerometer	Spain	Good
Huffman et al. [47]	Cross-sectional / case–control	RA (51)	55 (46,64)	36	RT3 triaxial accelerometer	USA	Good
Hugo et al. [48]	Observational	RA (57)	57.6 ± 10.2	73%	Sensewear armband accelerometer	France	Good
Hurkmans et al. [49]	Multi-centre Cross -sectional	RA (271)	62 ± 14	178	SQUASH	The Netherlands	Good
Iverson et al. [50]	Longitudinal	RA (573)	61 ± 12	478	NHSPAQ II	USA	Good
Katz et al. [51]	Cross-sectional	RA (158)	59.2 ± 11.3	118	IPAQ	USA	Good
Khoja et al. [52]	Cross-sectional	RA (98)	58 ± 9	83	Sensewear armband (SWA) biaxial accelerometer	USA	Good
Knittle et al. [53]	Multicentre longitudinal	RA (129)	60.5 ± 13.6	88	SQUASH	The Netherlands	Fair
Lee et al. [54]	Cross-sectional	RA (176)	55 (range 23–86)	146	GT1M Actigraph	USA	Fair
Løppenthin et al. [55]	Cross-sectional	RA (443)	60 (range 21–88)	356	Leisure time physical activity questionnaire & PAS 2	Denmark	Good
Lundgren et al. [56]	Cross-sectional	RA (95)	60 ± 11.9	71	PAI	Sweden	Fair
Malm et al. [57]	Longitudinal Observational	RA (1387)	65 ± 15	967	Customized Questionnaire	Sweden	Good
Mancuso et al. [58]	Longitudinal / case-control	RA (122)	49 ± 12	84%	PAEI	USA	Good
McKenna, et al., [59]	Cross-sectional	RA (75)	20% ≤ 49, 20% 50–59, 40% 60–69, 20% 70–79	48	The SeanseWear (Pro 3 Armband (SWA)	Ireland	Good
Metsios et al. [60]	Cross-sectional	RA (244)	62.1 (IQR 53.8–69.4)	174	IPAQ	UK	Good
Mochizuki et al. [61]	Cross-sectional	RA (137)	64 ± 11.6	111	Liferecorder	Japan	Good
Munsterman et al. [62]	Cross-sectional	RA (60)	51.8 ± 10.4	44	SQUASH	The Netherlands	Good
Neuberger et al. [63]	Randomized intervention study	RA (220)	55.5 (range 40–70)	82.7%	Self-report: mean mins aerobic exercise / week + mean of other aerobic at T2/3	USA	Poor
Piva et al. [64]	Cross-sectional	RA (47)	56.5 ± 7	47	SenseWear Professional v 6.1 activity monitor	USA	Good
Prioreschi et al. [65]	Case-control / cross-sectional	RA (50)	48 ± 13	50	Hip-worn Actical accelerometer	South Africa	Good
Rongen-van Dartel et al. [66]	Cross-sectional	RA (167) Low: 42 High: 125	Low: 56.78 ± 11.03, High: 54.79 ± 10.59	Low: 33, High: 67	Ankle-worn actometer (Actilog version 4.1)	The Netherlands	Good
Semanik et al. [67]	Cross-sectional	RA (185)	70 (range 60–88)	185	Yale PA survey	USA	Fair
Stavropoulos-Kalinoglou et al. [68]	Cross-sectional	RA (150)	F: 59 (IQR 55–64) M: 60 (IQR 59–64)	102	IPAQ	UK	Good
Tierney et al. [69]	Cross-sectional	RA (59)	60.10 ± 11.27	41	Sensewear armband	Ireland	Good
Uutela et al. [70]	Cross-sectional	RA (200)	AO: 59 ± 11 No AO: 60 ± 13	153	The FIT Index of Kasari	Finland	Good
Van den Berg et al. [71]	Observational	RA (252)	60.5 ± 11.5	182	Customized self-report	The Netherlands	Fair
Van der Goes et al. [72]	Cross-sectional	RA (165)	Inactive: 57 ± 12 Insufficient: 57 ± 12 Recommended: 60 ± 11	71%	SQUASH	The Netherlands	Good

Age presented as mean or median (standard deviation, confidence interval, inter quartile range or range). Sex presented as number or %. *RA* Rheumatoid Arthritis*, OA* Osteoarthritis, *IPAQ* International Physical Activity Questionnaire, *IPAQ-SF* International Physical Activity Questionnaire – short form, *ESAI* Exercise Stage Assessment Instrument, *PADS* Physical Activity and Disability Survey, *PAFQ* Physical Activity Frequency Questionnaire, *SQUASH* Short Questionnaire to Assess Health-Enhancing physical activity, *NHSPAQ II* Nurses Health Study II Physical Activity Questionnaire, *PAS 2* Physical Activity Scale, *PAI* Physical Activity Index, *PAEI* Paffenbarger Physical Activity and Exercise Index, *FIT Index* Frequency Intensity Time Index, *AO* abdominal obesity

**Table 2:** Description of included SpA studies

Citations	Design	Participants (*n*)	Age	Sex (F)	PA measure	Location	Methodological Quality
Arends et al. [73]	Cross-sectional / observational	AS (115)	44.6 ± 12.1	44	IPAQ, SQUASH, and ActiGraph (GT1M)	The Netherlands	Good
Brodin et al. [74]	Observational	AS (50)	51.5 (22–76)	16	Customized scale	Sweden	Fair
Brophy et al. [75]	Cohort study	AS (326)	55 ± 14	21%	IPAQ-SF	Wales	Good
Fabre et al. [76]	Cross-sectional	axSpA (203)	46 ± 11.6	95	IPAQ	France	Good
Fongen et al. [77]	Cross-sectional / case control	AS (149)	49.3 ± 11.1	39%	IPAQ	Norway	Good
Haglund et al. [78]	Cross-sectional	SpA (2167)	55 ± 14	52%	Customized	Sweden	Good
Meesters et al. [79]	Cross-sectional	SpA (2167)	55.4 ± 13.9	52%	Customized	Sweden	Good
O’Dwyer et al. [80]	Case-control / cross-sectional	AS (39)	40 ± 9	7	RT3 triaxial accelerometer	Ireland	Good
Prince et al. [81]	Multi-centre retrospective observational	AS (52)	44.8 ± 13.2	7	Customized interview	Australia	Fair
Van Genderen et al. [82]	Case control / multi-centre cross-sectional	AS (135)	51 ± 13	54	Actigraph GT3X triaxial accelerometer	The Netherlands	Good
Van Genderen et al. [83]	Case control / cross-sectional	AS (24)	48 ± 11	10	Tracmor triaxial accelerometer and Baecke	The Netherlands	Good

Age presented as mean or median (standard deviation, confidence interval, inter quartile range or range). Sex presented as number or %. *AS* Ankylosing Spondylitis, *axSpA* Axial Spondyloarthritis, *SpA* Spondyloarthritis, *IPAQ* International Physical Activity Questionnaire, *IPAQ-SF* International Physical Activity Questionnaire – short form, *SQUASH* Short Questionnaire to Assess Health-Enhancing physical activity

**Table 3:** Summary of study results and pooled associations – Rheumatoid arthritis

Variable	Positive Relationship	Negative Relationship	No Relationship	Assoc	% Studies
Sociodemographics					
Age	55	35, 36, 40, 45, 49, 50, 53, 54^bc^, 61^c^, 64^ac^, 64^ac^, 65^ac^, 67^a^, 69^c^, 71, 72	33, 35, 39^a^, 41, 43, 46, 46^c^, 47^c^, 57, 59^c^, 62, 63, 66^c^	??	16/30 53%
Gender (female)	49, 57, 71	35, 35, 40, 47^c^, 55, 59^c^, 62, 66^c^, 69^c^	33, 36, 41, 43, 54^bc^, 63, 72	??	9/19 47%
Race /Ethnicity (Caucasian)	50, 54^bc^		33, 43, 47^c^, 67^a^	00	2/6 33%
Educational level	36, 49, 50, 64^ac^, 64^ac^, 71		35, 43, 54^bc^, 63, 67^a^	??	6/11 55%
Employment status	50, 67^a^, 69^c^		49, 59^c^, 71	?	3/6 50%
Income	36		35, 35, 67^a^	0	1/4 25%
Marital status	50		64^ac^, 64^ac^, 67^a^	0	1/4 25%
Living status (children, location)	36	36	35, 49, 59^c^, 71	00	1/6 17%
Smoking		51^b^, 69^c^	33, 39^a^, 59^c^, 71	00	2/6 33%
Any use of alcohol	50			+	1/1 100%
Language Comprehension	36		35	?	1/2 50%
Physical					
RA Duration		48^c^, 48^c^, 49, 64^ac^, 65^ac^, 61^c^, 72	33, 35, 35, 36, 39^a^, 41, 44^c^, 45, 46, 46^c^, 47^c^, 50, 54^bc^, 57, 63, 64^ac^, 66^c^, 67^a^	00	7/25 28%
Body Mass Index (BMI) / weight / obesity	61^c^	50, 51^b^, 52^c^, 52^c^, 54^bc^, 65^ac^, 66^c^, 68	33, 39^a^, 43, 46, 46^c^, 47^c^, 59^c^, 64^ac^, 64^ac^, 69^c^, 71, 72	??	8/21 38%
Comorbidities		36, 47^c^, 64^ac^	35, 35, 43, 50, 54^bc^, 63, 64^ac^	00	3/10 30%
Disease activity		33, 34, 45, 46, 47^c^, 49, 51^b^, 52^c^, 55, 59^c^, 61^c^, 69^c^, 72	39^c^, 40, 41, 44^c^, 46, 46, 46^c^, 46^c^, 46^c^, 48^c^, 48^c^, 50, 52^c^, 59^c^, 64^ac^, 64^ac^, 65^ac^, 66^c^	??	13/31 42%
Tender and swollen joint count			40, 40, 46, 46, 46^c^, 46^c^, 57, 57, 63, 66^c^, 66^c^	00	0/11 0%
Radiographic joint damage		72	44^c^, 57	0	1/3 33%
Aerobic fitness	62		40, 63	0	1/3 33%
Strength and muscle function	51^b^, 64^ac^		40, 41, 63, 64^ac^	00	2/6 33%
Range of motion			40, 41	0	0/2 0%
Balance	64^ac^		40, 41, 64^ac^	0	1/4 25%
Function (inability)		35, 36, 44^c^, 46, 46^c^, 47^c^, 50, 51^b^, 52^c^, 52^c^, 55, 59^c^, 59^c^, 61^c^, 64^ac^, 64^ac^, 65^ac^, 69^c^	34, 35, 39^a^, 40, 41, 42^c^, 43, 45, 49, 57, 57, 66^c^	− −	18/30 60%
Gait Speed	64^ac^, 64^ac^			+	2/2 100%
Pain		36, 53, 55, 61^c^	34, 35, 35, 40, 41, 43, 45, 47^c^, 54^bc^, 57, 62, 63, 66^c^	00	4/17 24%
Fatigue		36, 45, 50, 51^b^, 55, 55, 55, 55, 58, 63, 66^c^	34, 35, 35, 46, 46^c^, 55, 62	− −	11/18 61%
Sleep (good/high)	51^b^, 59^c^		55, 59^c^, 66^c^	?	2/5 40%
Stiffness		34		−	1/1 100%
Other (physical)					
Waist circumference		70	39^a^, 47^c^	0	1/3 33%
Waist to hip ratio		33	33, 46, 46^c^	0	1/4 25%
Abdominal obesity		70, 72		−	2/2 100%
Fat Mass Index			39^a^	0	0/1 0%
Lean/fat mass	51^b^			+	1/1 100%
Body fat		68		−	1/1 100%
Diabetes		72	33, 55, 66^c^	0	1/4 25%
Fat Free Mass Index			39^a^	0	0/1 0%
Plasma glucose/insulin		39^a^		−	1/1 100%
Total cholesterol			33, 39^a^	0	0/2 0%
Cholesterol efflux capacity			33	0	0/1 0%
Low-density lipoprotein			33, 33, 33, 33, 39^a^, 52^c^, 52^c^	00	0/7 0%
High-density lipoprotein	33, 33, 33, 33, 39^a^, 52^c^		33, 33, 52^c^	+ +	6/9 67%
Tryglycerides		52^c^, 72	33, 52^c^	?	2/4 50%
Dyslipidaemia		72		–	1/1 100%
Apolipoprotein A1	39^a^			+	1/1 100%
Apolipoprotein B			39^a^	0	0/1 0%
Oxidized LDL			39^a^	0	0/1 0%
Antibodies against phosphorylcholine	39^a^			+	1/1 100%
Anti-citrullinated protein antibodies			34, 72	0	0/2 0%
Erythrocyte sedimentation rate			34, 39^a^, 40, 46, 46^c^, 47^c^, 57, 61^c^, 63, 65^ac^, 66^c^	00	0/11 0%
C-reactive protein		33, 61^c^	34, 40, 46, 46^c^, 51^b^, 59^c^, 59^c^, 63, 65^ac^, 66^c^, 72	00	2/13 15%
Matrix metalloproteinase (MMP-3)			61^c^	0	0/1 0%
RF positive			34, 66^c^	0	0/2 0%
Cardiovascular risk / disease		46^c^, 72	46, 46, 46^c^, 66^c^	00	2/6 33%
Hypertension		33, 72		−	2/2 100%
Systolic blood pressure		52^c^, 52^c^	33	−	2/3 67%
Diastolic blood pressure		52^c^, 52^c^	33	−	2/3 67%
Heart rate		51^b^	33	?	1/2 50%
Metabolic syndrome		46, 72	46^c^	−	2/3 67%
Nutritional complications		48^c^, 48^c^		−	2/2 100%
Augmentation Index		33		−	1/1 100%
Pulse wave velocity		33	33	?	1/2 50%
Agatson Score			33	0	0/1 0%
Insulin resistance		33, 52^c^, 52^c^		−	3/3 100%
RA -related joint surgery		50		−	1/1 100%
COPD			66^c^	0	0/1 0%
Haemoglobin			66^c^	0	0/1 0%
Psychological					
Exercise beliefs and (outcome) expectations	36, 36, 38^c^, 54^bc^, 63		35, 35, 35, 35, 43	??	5/10 50%
Motivation	38^c^, 47^c^, 49, 53, 54^bc^		47^c^	+ +	5/6 83%
Subjective Vitality	42^c^			+	1/1 100%
Self-efficacy	35, 36, 43, 47^c^, 47^c^, 50, 53		35, 63, 66^c^	+ +	7/10 70%
Depression / anxiety		42^c^, 51^b^	35, 35, 55, 62, 63, 66^c^	00	2/8 25%
Life worries			38^c^	0	0/1 0%
Health perception (good global health / assessment)	34, 36, 50, 50, 55, 61^c^		34, 35, 35, 40, 41, 57, 59^c^, 59^c^	??	6/14 43%
Fear Avoidance Beliefs		36, 37	35, 35, 56	?	2/5 40%
Other (psychological)					
Life beliefs			63	0	0/1 0%
Health locus of control			41, 56	0	0/2 0%
Pain and impairment relationship			56	0	0/1 0%
Health-related quality of life	50, 53, 53, 65^ac^		46, 46, 46^c^, 46^c^, 54^bc^, 65^ac^	??	4/10 40%
Individual SF-36 measures (health status)		34, 34, 50	44^c^, 44^c^, 44^c^, 44^c^, 44^c^, 44^c^, 44^c^, 44^c^, 65^ac^, 65^ac^, 65^ac^, 65^ac^, 65^ac^, 65^ac^, 65^ac^, 65^ac^, 65^ac^, 66^c^, 66^c^, 66^c^	00	3/23 13%
Arthritis Impact			34	0	0/1 0%
Beliefs about causes of fatigue			66^c^, 66^c^	0	0/2 0%
Coping strategies (fatigue)			66^c^, 66^c^, 66^c^, 66^c^, 66^c^, 66^c^	00	0/6 0%
Fatigue catastrophizing			66^c^, 66^c^, 66^c^	0	0/3 0%
Social					
Social support	36, 42^c^	35	35, 35, 35, 43, 49, 63	00	2/9 22%
Other					
Previous levels of PA	35, 35, 41			+	3/3 100%
Medications / biologics		46, 46^c^, 72	39^a^, 39^a^, 39^a^, 47^c^, 47^c^, 47^c^, 47^c^, 50, 50, 50, 50, 50, 51^b^, 66^c^, 66^c^, 66^c^, 66^c^, 72, 72	00	3/22 14%
Hospital admission /length		60, 60		−	2/2 100%
Goal Achievement			53	0	0/1 0%

^a^Female participants only

^b^Studies that investigated physical inactivity

^c^Associations from objective measures of PA

In the associations column the following applies: +  = positive association; −= negative association; ? = indeterminant/inconsistent; 0 = no association. If four or more studies indicate the same association the codes are +  + , − −, ?? and 00. The code is based on the percentage of studies supporting an association: 0–33% = 0; 34–59% = ?; and 60–100% =  + or −

**Table 4:** Summary of study results and pooled associations—Spondyloarthritis

Variable	Positive relationship	Negative relationship	No Relationship	Assoc	% Studies
Sociodemographics					
Age	78, 83	78	74, 76, 82^c^, 83^c^	00	2/7 29%
Gender (female)	78	78	74, 76, 82^c^, 82^c^	00	1/6 17%
Marital status (married)		74	76	?	1/2 50%
Employment (employed)			74, 76	0	0/2 0%
Educational level			76, 78, 78	0	0/3 0%
Raised children under 12 years			76	0	0/1 0%
Smoking		78	78	?	1/2 50%
Physical					
Comorbidities			76	0	0/1 0%
Symptom duration			74, 76	0	0/2 0%
Diagnosis duration	74	82^c^	82^c^, 83, 83^c^	0	1/5 20%
Peripheral Joint involvement			74	0	0/1 0%
Disease activity	74, 76, 82^c^	73, 73, 73, 73, 73^c^, 75, 77, 78	73^c^, 76, 78, 80^c^, 80^c^, 82^c^, 83, 83^c^	??	8/19 42%
Erythrocyte sedimentation rate		73^c^	73, 73	0	1/3 33%
C-reactive protein		73^c^	73, 73, 76	0	1/4 25%
Function (inability)	74	73, 73, 73^c^, 75, 78, 78, 82^c^, 83^c^	76, 80^c^, 82^c^, 83	− −	8/13 62%
Spinal immobility			74	0	0/1 0%
Occiput-to-wall distance		73	73, 73^c^	0	1/3 33%
Chest expansion			73, 73, 73^c^	0	0/3 0%
Modified Schober test	73, 73^c^		73	+	2/3 67%
Lateral spinal flexion	73^c^		73, 73	0	1/3 33%
Cervical rotation	73, 73^c^		73	+	2/3 67%
Radiographic signs			76	0	0/1 0%
Axial pain			76	0	0/1 0%
Fatigue		83, 83, 83	83, 83, 83^c^, 83^c^, 83^c^, 83^c^, 83^c^	00	3/10 30%
Psoriatic arthritis subtype		78	78	?	1/2 50%
Undifferentiated spondyloarthritis subtype			78, 78	0	0/2 0%
Inflammatory bowel disease-related arthritis			78, 78	0	0/2 0%
Uveitis			76	0	0/1 0%
Psoriasis			76	0	0/1 0%
Inflammatory bowel disease			76	0	0/1 0%
HLA B27 antigen			76	0	0/1 0%
Inflammatory back pain			76	0	0/1 0%
Arthritis			76	0	0/1 0%
Enthesitis			76	0	0/1 0%
Dactylitis			76	0	0/1 0%
Surgery due to axSpA			76	0	0/1 0%
Body Mass Index (BMI)		82^c^	76, 82^c^, 83, 83^c^	00	1/5 20%
Aerobic fitness (VO_2_max)	80^c^			+	1/1 100%
Having ankylosing spondylitis		81		−	1/1 100%
Psychological					
Health perceptions (overall / disease)		74	74, 76, 83, 83^c^	00	1/5 20%
Quality of life	73, 73, 73^c^, 78, 78		80^c^	+ +	5/6 83%
Motivation	75, 75, 75	75	75, 75	?	3/6 50%
Perception of exercise			76, 76	0	0/2 0%
Depression		79	76, 78, 78	0	1/4 25%
Anxiety			76, 78, 78, 79	00	0/4 0%
Environmental					
Seasonal variation (summer)	77			+	1/1 100%
Other					
Previous levels of PA	74			+	1/1 100%
Medication / biologics	81		74, 76, 76	0	1/4 25%
Age of anti-TNF therapy		81		−	1/1 100%
Physiotherapy			76	0	0/1 0%

^c^Associations from objective measures of PA

In the associations column the following applies: +  = positive association; −= negative association; ? = indeterminant/inconsistent; 0 = no association. If four or more studies indicate the same association the codes are +  + , − −, ?? and 00. The code is based on the percentage of studies supporting an association: 0–33% = 0; 34–59% = ?; and 60–100% =  + or −

### Measurement of PA

Self-reported measures of PA were used in twenty-seven RA studies. Twenty-one RA studies used known self-report questionnaires, whereas six studies applied custom questionnaires or interviews. Objective measures of PA were used in fourteen RA studies. Thirteen RA studies used an accelerometer, and one used a Liferecorder. Nine SpA studies used self-reported measures of PA, whereby, five SpA studies used validated questionnaires, and a further four used customized scales. Objective measures of PA were used in four SpA studies (i.e., accelerometers).

### Correlates of PA in rheumatoid arthritis

#### Sociodemographic

The most frequently studied sociodemographic variables were age and gender. Across studies, age and gender showed inconsistent associations with PA, yet the direction of associations were largely split between inverse (age, 53%; female gender, 47%) or no relationship. Higher educational level and employment status were indeterminately associated with PA parameters; however, over half of the associations were reported as positive (educational level, 55%; and employment status, 50%). Non-significant associations were found for race/ethnicity, income, marital status, living status, and smoking.

#### Physical

Consistent negative associations with PA were reported for functional disability (60%) and fatigue (61%). However, Løppenthin et al. [55] utilized the five domains of the Multidimensional Fatigue Inventory (MFI-20 [84]), contributing four negative and one non-significant association to the pooling. Removing this study would categorise fatigue as inconsistently related to PA parameters, with only 54% of associations negative. Although disease activity and body mass index (BMI) received inconsistent summary codes, 42% and 38% of associations were negative. Disease activity was measured using the Disease Activity Score 28 (DAS28) in 19 studies, the Simplified Disease Activity Index (SDAI) in 2 studies, and the Rheumatoid Arthritis Disease Activity Index (RADAI) in 2 studies (based on included associations). The pooled associations between sleep and PA were indeterminant, with two associations supporting a positive relationship (40%). Non-significant associations with PA were consistently reported for RA duration, comorbidities, tender and swollen joint count, strength and muscle function, and pain.

Of the biochemical variables, HDL was consistently positively related to PA parameters (67%), whereas low-density lipoprotein (LDL) was unrelated. Additional variables demonstrating consistent and non-significant associations with PA parameters include erythrocyte sedimentation rate (ESR), C-reactive protein (CRP), and cardiovascular risk.

#### Psychological

In adults with RA, motivation was consistently and positively related to PA in 5 of the 6 comparisons (83%), in addition to, self-efficacy in 7 of 10 comparisons (70%). Motivation was assessed in 5 studies, using a variety of scales (Customized scale based on the Perceived Competence Scale, *n* = 1; an adapted scale (for endurance and strength training motivation) previously used in those with arthritis, *n* = 1; the Treatment Self-Regulation Questionnaire (TSRQ), *n* = 2; and a 6-item scale for assessing motivation for increasing PA (confidence to maintain an active lifestyle) based on the Interaction Model of Client Health Behavior (IMCHB), *n* = 1). Self-efficacy was assessed in 8 studies, also using a range of assessment tools (Exercise Self-Efficacy Scale (ESES), *n* = 2; Arthritis Self-Efficacy Scale (ASES), *n* = 1; an adapted scale (for endurance and strength training self-efficacy) previously used in those with arthritis, *n* = 1; Lorig’s Self Efficacy Scale (for managing arthritis), *n* = 1; goal self-efficacy subscale of the Self-Regulation Skills Battery, *n* = 1; the mean of two confidence measures, *n* = 1; and the Self-Efficacy Scale 28 (for fatigue), *n* = 1). One study investigated subjective vitality, whereby a positive correlation with light PA was found (*r* = 0.27, *p* < 0.05) [42]. Positive associations were seen for exercise beliefs and outcome expectations (50%), health perception (43%) and health-related quality of life (40%), yet these resulted in inconsistent summary codes. The psychological variables most consistently reporting non-significant associations with PA were depression/anxiety (75%), coping strategies for fatigue (100%) and the individual components of the 36-Item Short Form Survey (SF-36; 87%). Studies utilizing the individual components of the SF-36 were grouped due to the disparity of studies reporting total quality of life versus the individual components.

#### Social

The relationship between social support and PA parameters were examined in six studies. Within these six studies, 6 of 9 associations between social support and PA were non-significant (67%).

#### Environmental

Environmental variables were not examined in any of the included RA studies.

#### Other

Three associations from two studies (100%) found that already established PA at baseline was a significant positive predictor of PA. Eurenius et al. [41] found that high baseline PA predicted high levels of PA after one year (OR 3.85, 95% CI 1.67–9.09). Similarly, Demmelmaier et al. [35] identified three trajectories of PA over two years, and previously established PA was a significant positive predictor of being in the stable high vs the decreasing or stable low trajectory. Further, negative associations were reported in one study between PA and the number and length of hospital admissions [60]. Medications and biological treatment were not significantly related to PA parameters. Negative associations were found for corticosteroid use [46] and glucocorticoid therapy [72].

#### Pooled associations based on most adjusted or final model statistics

Online Resource 1 shows the pooled associations when considering the final model and adjusted associations for each variable. When pooled in this fashion, the indeterminant relationships of employment status shifted to unrelated. Further, the positive relationships of alcohol use and gait speed changed to unrelated. The negative relationships of functional disability, abdominal obesity, nutritional complications, and hypertension shifted to inconsistent. Fatigue was negatively related to PA parameters, yet when pooling for the most adjusted or final models this relationship changed to unrelated. This association change remained the same when removing the results reported by Løppenthin et al. [55] which contributed five independent domains of fatigue. The inconsistent pooled results for disease activity, triglycerides, health perception, fear avoidance beliefs, and health-related quality of life all shifted to unrelated.

#### Pooled associations with only objective measures of PA

The main differences between the pooled associations from all studies and the pooled associations from studies using objective measures of PA are as follows (all studies vs objective PA studies): age (16/30, 53%, ?? vs. 6/10, 60%, − −); gender (9/19, 47%, ?? vs. 4/5, 80%, − −); race/ethnicity (2/6, 33%, 00 vs. 1/2, 50%, ?); educational level (6/11, 55%, ?? vs. 2/3, 67%, +); smoking (2/6, 33%, 00 vs. 1/2, 50%, ?); RA duration (7/25, 28%, 00 vs. 5/11, 45%, ??); comorbidities (3/10, 30%, 00 vs. 2/4, 50%, ?); disease activity (13/31, 42%, ?? vs. 5/18, 28%, 00); strength and muscle function (2/6, 33%, 00 vs. 1/2, 50%, ?); balance (1/4, 25%, 0 vs. 1/2, 50%, ?); fatigue (11/18, 61%, − − vs. 1/2, 50%, ?); sleep (2/5, 40%, ? vs. 1/3, 33%, 0); metabolic syndrome (2/3, 67%, − vs. 0/1, 0%, 0); HDL (6/9, 67%, ++ vs. 1/2, 50%, ?); exercise beliefs and outcome expectations (5/10, 50%, ?? vs. 2/2, 100%, +); depression / anxiety (2/8, 25%, 00 vs. 1/2, 50%, ?); health perception (6/14, 43%, ?? vs. 1/3, 33%, 0); social support (2/9, 22%, 00 vs. 1/1, 100%, +). Some variables were only assessed in studies using self-reported measures of PA and therefore comparisons cannot be made (e.g., income, fear avoidance beliefs).

### Correlates of PA in Spondyloarthritis

#### Sociodemographic

In adults with SpA, none of the sociodemographic variables showed consistent positive or negative associations with PA. Marital status and smoking were negatively related to PA in 1 of 2 associations (50%), resulting in inconsistent summary scores. Age and gender were consistently not associated with PA; however, in two studies (29%) and one study (17%) respectively, positive associations were found. Some evidence suggested that variables such as employment status, education and raising children under 12 years old, were also unrelated to PA.

#### Physical

The two most frequently studied physical variables were functional disability and disease activity. Functional disability was the only physical variable that was consistently and negatively associated with PA parameters (62% of 13 associations). The summary score between disease activity and PA was deemed indeterminant, however, 42% of 19 associations were negative. Disease activity was assessed using the Bath Ankylosing Spondylitis Disease Activity Index (BASDAI) in 8 studies and the Ankylosing Spondylitis Disease Activity Score (ASDAS-CRP) in 4 studies (based on included associations). One study utilized the total Bath Ankylosing Spondylitis Metrology Index (BASMI) score as an indicator of spinal immobility and found it unrelated with PA [74]. However, Arends et al. [73] investigated the separate components of spinal mobility in relation to PA using three different measures of PA (International Physical Activity Questionnaire (IPAQ), Short QUestionnaire to ASsess Health-enhancing physical activity (SQUASH) and Actigraph, GT1M). The Modified Schober test and cervical rotation were positively related to PA in 2 of 3 (67%) comparisons. One positive association was found between lateral spinal flexion and accelerometer measured PA (33%), whereas one negative association was reported between occiput-to-wall distance and PA measured using the SQUASH (33%). Chest expansion was unrelated to PA parameters. The direction of these associations indicates that PA is positively related to spinal mobility. In one study [83], negative associations were seen for reduced activity, mental and physical fatigue (30%), however other sub-categories of the MFI-20-G were non-significant (70%).

In one study, SpA subtypes such as inflammatory bowel disease (IBD)-related arthritis and uSpA were not significantly associated with meeting WHO MI-PA or VI-PA recommendations, however, one negative relationship (OR 0.72, 95% CI 0.56–0.92, *p* = 0.009) between the diagnosis of PsA and meeting the VI-PA WHO recommendations was found [78]. Extra-musculoskeletal manifestations, BMI, ESR and CRP were also unrelated to PA.

#### Psychological

Of the psychological variables, only higher quality of life was consistently and positively related to increased PA parameters (83%) in adults with SpA. Although motivation was only investigated in one study, the indeterminant summary code is not overly representative of the findings from this study. Brophy et al. [75] investigated motivation using the Behavioural Regulation in Exercise Questionnaire (BREQ-2) and found relationships between motivation, individual motivation regulations and PA. Positive associations between intrinsic (*β* slope 1320 (960–1680), *p* < 0.05) and identified motivation (*β* slope 994 (651.5–1336), *p* < 0.05) and PA were found. Further, introjected motivation and external motivation were unrelated to PA, while amotivation (*β* slope − 667 (− 1242 to − 92), *p* < 0.05) was negatively related. The findings by Brophy et al. [75] are consistent with the tenets of self-determination theory showing that autonomous motivations towards exercise had the greatest influence on PA levels [85]. Thus, moving forward, motivation will be discussed as being positively related to PA in those with SpA. Health perceptions, depression, and anxiety were unrelated to PA, yet the associations were derived from only three studies.

#### Social

Social variables were not examined in any of the included SpA studies.

#### Environmental

Seasonal variation was the only environmental factor explored. One cross-sectional comparative study [77] showed a positive relationship (*p* ≤ 0.02) between total weekly PA energy expenditure and summer months (i.e., as compared to winter).

#### Other

In one study [74], results of multiple logistic regression analyses showed that previous levels of PA positively predicted PA after two years (OR 37.03, 95% CI 3.85–370.00, *p* = 0.002). Physiotherapy and the use of biologics or medication were unrelated to PA. Yet, one multicentre observational study limited to retrospective data, reported that age at onset of anti-Tumor Necrosis Factor alpha (anti-TNFα) therapy was negatively related to PA, whereas anti-TNFα use was associated with increased PA [81].

#### Pooled associations based on most adjusted or final model statistics

Considering the results of the pooled associations of PA based on the most adjusted or final models for each variable (see Online Resource 2), the association of employment status and symptom duration shifted (non-significant to indeterminant). Further, the indeterminant association of disease activity changed to unrelated.

#### Pooled associations with only objective measures of PA

The main differences between the pooled associations from all studies and the pooled associations from studies using objective measures of PA are as follows (all studies vs objective PA studies): disease activity (8/19, 42%, ?? vs. 1/7, 14%, 00); ESR (1/3, 33%, 0 vs. 1/1, 100%, −); CRP (1/4, 25%, 0 vs. 1/1, 100%, −); lateral spinal flexion (1/3, 33%, 0 vs. 1/1, 100%, +); and quality of life (5/6, 83%, +  + vs. 1/2, 50%, ?). Several variables were only measured in studies using self-reported measures of PA and therefore comparisons cannot be made (e.g., employment status, comorbidities, motivation, and previous levels of PA).

## Discussion

This systematic review provides a needed update of the multifaceted correlates of PA in RA, in a field advancing in pharmacological treatments and more frequently utilizing objective measures of PA. To the best of our knowledge, this is also the first systematic review to synthesise the correlates of PA among adults living with SpA. A total of 40 RA and 11 SpA studies were reviewed with correlates examined in the following domains: sociodemographic; physical; psychological; social; and environmental.

### Main correlates of PA—Rheumatoid Arthritis

The current systematic review extends and updates Larkin and Kennedy’s [24] review that was limited to ten studies with mainly cross-sectional designs (8/10) and only one study utilizing objective measures of PA. The pooled results of the RA studies in this review support those presented by Larkin and Kennedy [24]. Motivation, self-efficacy, and previous levels of PA were positively associated with PA parameters. Fatigue and plasma glucose were negatively related with PA. HDL was consistently positively related with PA parameters. However, the positive association of health perception with PA was indeterminant in this review. Additional positive associations identified via this latest review include, any use of alcohol, gait speed, lean fat/mass, apolipoprotein A1 and subjective vitality. Additional negative associations include functional inability, stiffness, abdominal obesity, body fat, hypertension, blood pressure, metabolic syndrome, nutritional complications, insulin resistance, RA-related joint surgery, and hospital admissions/length. The most frequently studied variables showing a lack of consistent findings include age, gender, educational level, BMI, disease activity, exercise beliefs, health perception, and health-related quality of life.

This review did not find any of the sociodemographic variables to be consistently related to PA in those with RA. Some evidence that any use of alcohol (100%, *n* = 1/1) was positively related to PA was reported, however, this was investigated in one study, and was unrelated to PA when the results where pooled based on the most adjusted or final models for each variable. Age and female gender were inconsistently related to PA; however, the associations were predominantly negative (age, 53%, *n* = 16/30; female gender, 47%, *n* = 9/19) and non-significant. The trend towards a negative relationship is somewhat consistent with findings in the general population of older adults, whereby higher age and female sex are associated with a decreased likelihood of persistent PA over ten years [86]. The reason for inconsistent or indeterminant pooled associations between sociodemographic variables and PA is likely to be multifaceted. For example, the age ranges between samples varies considerably, with studies utilizing both narrow (i.e., 40–70 years) [63] and wide (i.e., 19–90 years) [40] age ranges. Further, a gender imbalance is observed in many studies, with a female dominance of participants. Assessment of PA may also be a factor, as when pooling only associations of objective measures of PA, female gender was consistently and negatively related to PA (80%, n = 4/5). A possible reason for the negative association between female gender and PA could be that females report greater disease activity and physical function, compared to males with RA [87]. Race/ethnicity was consistently unrelated to PA parameters, yet, caution should be taken in the interpretation of this result, as racial or ethnic minorities were not proportionately represented in some studies. For example, one study reported that 71% of participants were Caucasian, whereas 2% were Asian, and 22% African American [47]. In another study of 573 individuals with RA, 94% of the participants were Caucasian [50].Of the physical variables, functional disability was the most consistent and negative correlate of PA parameters (60%, n = 18/30). Although the findings were more inconsistent when considering the most adjusted or final model statistics, this finding corroborates recent reports linking worse physical functioning to lower levels of PA in those with early RA [88] and PA maintenance over a 7-year period [89]. Further, fatigue was negatively related to PA (61%, n = 11/18), yet the results may be distorted due to one study [55] utilizing the five separate domains of the MFI-20. Univariately, Løppenthin et al. [55] found four of the five domains of fatigue to be inversely associated with regular PA. However, in the multivariate model, only reduced activity due to fatigue and physical fatigue remained significant. Predominantly, our findings support recent longitudinal studies showing a negative relationship between fatigue and PA [89, 90]. Interestingly, disease activity was inconsistently related to PA, however, almost half of the associations were negative (42%, n = 13/31). Disease activity was unrelated to PA when pooling the results based on the most adjusted statistics or when considering studies utilizing objective measures of PA. One possible reason for the inconsistency in results might relate to whether participants were experiencing a flare when PA was measured. Further, the relationship between disease activity and PA might be dependent on the contribution of other variables in explaining PA. For example, recent reports have shown no association between PA and disease activity, when controlling for age and fatigue [91]. BMI and obesity have been shown to negatively associate with PA in the general population [86] and those with hip osteoarthritis [92], yet our findings suggest this relationship is more inconsistent in people living with RA (38%, *n* = 8/21). This inconsistency in findings is not surprising considering the complex nature of energy expenditure, which is largely determined by body composition and body weight [93]. In support of a previous review [24], RA duration and pain was unrelated to PA parameters. The most consistent physiological variable positively related to PA parameters was HDL (67%, *n* = 6/9). Other physiological variables, especially those detecting inflammation (i.e., ESR and CRP), were unrelated to PA.

Self-efficacy was one of the most consistent psychological variables positively related with PA (70%, *n* = 7/10). Greater self-efficacy has been positively associated with PA in healthy populations [94] and women with other long-term conditions such as fibromyalgia [95]. This review adds to the evidence supporting the association between self-efficacy and PA previously reported by Larkin and Kennedy [24], with the addition of six more studies (seven associations) in the RA population. Inconsistent findings were reported for exercise beliefs and expectations, health perception (including global health/assessment), and health-related quality of life. Motivation was the most consistent psychological variable positively related to PA (83%, *n* = 5/6) in this review. This positive relationship is supported by studies highlighting the importance of autonomous motivation in predicting PA participation levels in 207 RA members of the National Rheumatoid Arthritis Society [96] and in increased engagement in moderate-to-vigorous PA (MVPA) during a randomized controlled trial [97]. Two studies investigating motivation regulation style (on the self-determination theory continuum), showed an autonomous regulatory style to be associated with higher PA, and a more controlling regulatory style to relate to lower PA. The importance of autonomous regulations in fostering PA behaviour is supported by the findings of a systematic review exploring the relationship between self-determination theory constructs (e.g., autonomous vs. controlled motivation) and PA [85].

Social variables in this review were limited to social support and no significant relationship was found. This non-significant relationship was surprising as research conducted with people living with knee and hip osteoarthritis, has reported that perceptions of autonomy support from their spouse regarding PA to be positively related to PA, while perceived pressure from a female spouse (to a male partner), has been shown to be negatively related to PA [92]. In the current review, positive (*n* = 2), negative (*n* = 1), and non-significant relationships (*n* = 6) were found between social support and PA parameters. The variability in results may be due to the differences in assessment methods (Social Support for Exercise Behaviors Scale, 2 studies; Important Other Climate Questionnaire, 1 study; Health Care Climate Questionnaire, 1 study; Medical Outcomes Study Social Support Survey, 2 studies), type of PA parameter (e.g., total PA, trajectory of PA, health-enhancing PA or LPA), or the type of individual assessed for social support (e.g., spouse, friend, rheumatologist). Further research is needed to explore this variability and the factors that affect the relationship between social correlates and PA behaviour.

No environmental correlates were investigated in the RA studies. Of the other variables, and in concordance with two other reviews [23, 24], previous levels of PA were shown to be positively associated with PA parameters (100%, *n* = 3/3).

### Main correlates of PA—Spondyloarthritis

In SpA studies, positive associations with PA were found for components of spinal mobility (Modified Schober test and cervical rotation), maximum volume of oxygen (VO2max), quality of life, motivation, previous PA and seasonal variation. However, in a model predicting PA after two years, spinal immobility (total BASMI) was not significant [74]. Negative associations with PA were found for functional disability, having AS, being amotivated, and age at onset of anti-TNFα therapy. Indeterminant associations were found for marital status, smoking, disease activity, and PsA subtype. All other variables were not significantly associated with PA parameters.

None of the sociodemographic variables were positively or negatively related to PA in those with SpA. Gender and age were unrelated to PA in this review. However, total self-reported PA, in 24 AS patients (aged 48 ± 11 [83]) and meeting the WHO MI-PA recommendations in 2,167 SpA patients (aged 55 ± 14 [78]) were positively associated with age in two studies (29%, *n* = 2/7). Marital status and smoking were inconsistently related to PA and employment status, education and children raised under 12 years old were unrelated in a limited number of studies.

Of the physical variables, functional disability was frequently investigated, with studies consistently supporting a negative correlation with PA parameters (62%, 8/13). This is supported by recent findings from a prospective cohort study which found that time spent in MVPA was associated with better function [98]. Surprisingly, spinal immobility, measured via total BASMI score was only reported in one study [74]. Here, a non-significant association with PA was reported. However, Arends et al. [73] reported on the relationship between PA and the separate domains of spinal mobility, and together, the direction of associations indicates that higher PA is related to better spinal mobility. This association has also been supported in relation to time spent in MVPA [98], yet the evidence from exercise programmes is more conflicting and lacks clarity [99]. Longitudinal studies are needed to help clarify the relationship between PA and spinal mobility, and the types of PA most related.

Mixed and inconsistent associations were found between PA and disease activity, with studies reporting positive (*n* = 3), negative (*n* = 8) and non-significant (*n* = 8) associations. Such disparity in findings is commensurate with a review investigating the correlates of PA in people with arthritis [23]. The inconsistency in results may partially be due to the intensity of PA measured, for example, one study showed that higher disease activity (measured via the BASDAI) was associated with greater LPA in adults with AS aged ≥ 52 years, but not associated with MVPA in AS patients across all ages [82]. A further explanation for the inconsistency in results may be that some individuals with SpA may participate in PA as a reaction to disease activity, in comparison to those who may start PA engagement earlier as a preventative measure against disease activity. Interestingly, only one study investigated whether SpA subtype was associated with PA [78]. In multivariate analyses, uSpA and IBD-related arthritis were not significantly associated with meeting the MI-PA or VI-PA recommendations, however, PsA was negatively related to meeting VI-PA recommendations. One possible explanation is that individuals with PsA typically experience more inflammation of the peripheral joints than other SpA subtypes [100] and have separate clinical presentations. Due to the limited number of studies, more research is needed to determine potential differences between SpA subtype and PA parameters.

Of the psychological variables, a self-reported better quality of life was associated with higher PA (83%, *n* = 5/6) in those with SpA. This association is supported in an online survey of 445 Danish individuals with SpA [101] and those with hip osteoarthritis [92]. Motivation to exercise was investigated in one study [75], whereby intrinsic motivation was positively related to PA levels. Using a path analysis, autonomous forms of motivation (i.e., intrinsic motivation and identified regulation) were positively associated to PA, whereas amotivation was negatively associated. Regulation style has also been found to be a significant determinant of PA in those with RA [49]. The importance of motivation is further highlighted in a behaviour change intervention for those with axial spondyloarthritis. Those undertaking the intervention aimed at supporting PA motivation had improved health-enhancing PA and spinal mobility [102]. Despite the apparent importance of motivation on PA behaviour [85], little research has been conducted in those with SpA. Future research should focus on longitudinal study designs or the utilization of ecological momentary assessment methods [103] to yield insight into the causal and fluctuating relationships between PA motivation and PA behaviour.

Previous levels of PA were found to positively correlate to PA [74]. Based on retrospective data, one study reported that individuals who started anti-TNFα therapy earlier had greater gains in PA [81]. Seasonal variation (summer) was positively associated with PA energy expenditure in one study [77].

## Recommendations for future research

Many studies adopted a cross-sectional design and therefore causal effects cannot be determined. To establish the causal relationships between variables and PA, prospective and interventional studies are required. In both RA and SpA studies, psychological variables (namely motivation) are underrepresented despite significant associations and the support of intervention studies. In those with SpA, negative and non-significant associations between mental and physical fatigue, and PA were identified, yet fatigue was only evaluated in one study. Research is needed to determine if a stable relationship between fatigue and PA exists in those with SpA. Self-efficacy is positively related to PA in those with RA, yet self-efficacy was not measured in any of the SpA studies. Studies investigating correlates of PA in the axSpA population was generally sparse. Only one eligible study, investigated diagnoses of SpA other than nr-axSpA and AS. The lack of data reported for more peripheral SpA subtypes (e.g., PsA), poses a challenge to clinicians aiming to support PA behaviour. Considering PA is highly recommended in those with SpA, a greater effort is needed to verify the determinants of PA and evaluate potential differences between subtypes. Researchers would do well to employ longitudinal and interventional studies to explore functional ability, in addition to supporting PA self-efficacy and autonomous motivation in these populations. Whilst the reasons for inconsistent results are likely to be multifaceted, the presence of flares have been shown to impact PA behaviour in those with RA and SpA [104]. Although difficult to measure, no eligible studies in this review utilized flares as a variable. Future research should incorporate flares as a variable, with the aim to provide clinically useful information regarding disease activity and its impact on PA.

## Strengths and limitations

This systematic review provides an up-to-date and valuable synthesis of the correlates of PA in those with RA and SpA, which can help inform clinical practitioners and intervention design to support the initiation and maintenance of PA. However, several strengths and limitations of this review are apparent. The systematic review included an array of variables, measures of PA (i.e., self-report and objective) and parameters of PA (e.g., total PA, meeting PA recommendations, MVPA). Multiple measures and parameters of PA allow for a more complete account of PA correlates but somewhat hinders the ability to define and conclude the strength of any relationship (i.e., via meta-analysis). A limited number of studies reporting potential correlates of PA in the SpA population met the inclusion criteria. This was somewhat alleviated by permitting studies that included a doctor diagnosis of SpA, instead of the having to meet a classification criterion. While this allows greater opportunity for the exploration of PA correlates in SpA studies, the uniformity between patients is less stringent than if a classification criterion was applied. RA studies were included if the study cohort met the ACR/EULAR criteria [26], which should be considered when comparing the RA and SpA studies and the potential correlates of PA identified. Further, ten SpA studies (*n* = 10/11) were conducted in Europe, therefore, it is difficult to extrapolate these results to SpA populations outside of Europe who may have differing living conditions, health systems and treatment options. The findings are more generalizable to female persons with RA and male persons with SpA. Female participants were represented more than males in most RA studies, while four RA studies included female participants only. Participation rate of eligible persons and sample size justification were often not reported. The review was limited to fourteen RA (*n* = 14/40) and four SpA studies (*n* = 4/11) that utilized objective measures of PA. The remaining studies employed self-report measures of PA, which increases the risk of bias. Self-reported measures of PA may be subject to recall bias, with over or under-reporting of activity possible [105, 106]. The ability to evaluate causal relationships was limited due to the large number of studies adopting a cross-sectional design. Two RA studies were assessed as having a ‘poor’ quality rating, due to unclear reporting [34] and a greater than 15% differential drop-out rate between intervention groups [63].

## Conclusions

The aim of this review was to identify potential and consistent correlates of PA in those living with RA and SpA. Few consistent associations with PA were found in the RA and SpA populations. In individuals with RA, consistent positive associations were found between PA and HDL, self-efficacy and motivation, and consistent negative associations with functional disability and fatigue. In individuals with SpA, consistent positive associations were found between PA and quality of life, and consistent negative associations with functional disability. Prospective studies investigating the correlates of PA in these populations are needed, especially in those with SpA. These findings should inform future research and the design of interventions aiming to promote PA in people with RA and SpA.

## Supplementary Information

Below is the link to the electronic supplementary material.

Supplementary file1 (DOCX 33 KB)

Supplementary file2 (DOCX 23 KB)

Supplementary file3 (DOCX 52 KB)
