# Supplementary material for: Correlates of physical activity in adults with spondyloarthritis and rheumatoid arthritis: a systematic review
Source: Rheumatol Int. 2022 Jun 8;42(10):1693–713. doi: 10.1007/s00296-022-05142-z (PMC9439989; doi:10.1007/s00296-022-05142-z)
Supplement: Supplementary file 1 — Supplementary file1 (DOCX 33 KB) [file 296_2022_5142_MOESM1_ESM.docx]

Article title: Correlates of physical activity in adults with spondyloarthritis and rheumatoid arthritis: a systematic review

Journal: Rheumatology International

Thomas Ingram^1, 2*^, Raj Sengupta^2, 3^, Martyn Standage^1^, Rosie Barnett^1,2^ and Peter Rouse^1^

^1^ Department for Health, University of Bath, Bath, UK; ^2^ Royal National Hospital for Rheumatic Diseases, Royal United Hospitals NHS Foundation Trust, Bath, UK; ^3^ Department of Pharmacy & Pharmacology, University of Bath, Bath, UK

*Corresponding author: Thomas Ingram. tai23@bath.ac.uk

**Supplementary Table 1** Summary of study results based on the most adjusted or final model statistics for each variable – Rheumatoid arthritis

| Variable | Positive Relationship | Negative Relationship | No Relationship | Assoc. | % Studies |
| --- | --- | --- | --- | --- | --- |
| Sociodemographics |  |  |  |  |  |
| Age | 36 | 35, 40, 45, 49, 50, 53, 54^bc^, 55, 61^c^, 64^ac^, 64^ac^, 65^ac^, 67^a^, 69^c^, 71, 72 | 33, 35, 39^a^, 41, 43, 46, 46^c^, 47^c^, 57, 59^c^, 62, 63, 66^c^ | ?? | 16/30 53% |
| Gender (female) | 71 | 35, 35, 40, 47^c^, 55, 59^c^, 62, 66^c^, 69^c^ | 33, 36, 41, 43, 49, 54^bc^, 57, 63, 72 | ?? | 9/19 47% |
| Race /Ethnicity (Caucasian) | 50, 54^bc^ |  | 33, 43, 47^c^, 67^a^ | 00 | 2/6 33% |
| Educational level | 36, 64^ac^, 64^ac^, 71 |  | 35, 43, 49, 50, 54^bc^, 63, 67^a^ | ?? | 4/11 36% |
| Employment status | 67^a^, 69^c^ |  | 49, 50, 59^c^, 71 | 00 | 2/6 33% |
| Income |  |  | 35, 35, 36, 67^a^ | 00 | 0/4 0% |
| Marital status |  |  | 50, 64^ac^, 64^ac^, 67^a^ | 00 | 0/4 0% |
| Living status (children, location) |  |  | 35,36, 36, 49, 59^c^, 71 | 00 | 0/6 0% |
| Smoking |  | 51^b^, 69^c^ | 33, 39^a^, 59^c^, 71 | 00 | 2/6 33% |
| Any use of alcohol |  |  | 50 | 0 | 0/1 0% |
| Language Comprehension | 36 |  | 35 | ? | 1/2 50% |
| Physical |  |  |  |  |  |
| RA Duration |  | 64^ac^, 65^ac^ | 33, 35, 35, 36, 39^a^, 41, 44^c^, 45, 46, 46^c^, 47^c^, 48^c^, 48^c^, 49, 50, 54^bc^, 57, 61^c^, 63, 64^ac^, 66^c^, 67^a^, 72 | 00 | 2/25 8% |
| Body Mass Index (BMI) / weight / obesity | 61^c^ | 50, 51^b^, 52^c^, 52^c^, 65^ac^, 66^c^, 68, 72 | 33, 39^a^, 43, 46, 46^c^, 47^c^, 54^bc^, 59^c^, 64^ac^, 64^ac^, 69^c^, 71 | ?? | 8/21 38% |
| Comorbidities |  | 47^c^, 64^ac^ | 35, 35, 36, 43, 50, 54^bc^, 63, 64^ac^ | 00 | 2/10 20% |
| Disease activity |  | 33, 34, 46, 47^c^, 51^b^, 55, 59^c^, 72 | 39^a^, 40, 41, 44^c^, 45, 46, 46, 46^c^, 46^c^, 46^c^, 48^c^, 48^c^, 49, 50, 52^c^, 52^c^, 59^c^, 61^c^, 64^ac^, 64^ac^, 65^ac^, 66^c^, 69^c^ | 00 | 8/31 26% |
| Tender and swollen joint count |  |  | 40, 40, 46, 46, 46^c^, 46^c^, 57, 57, 63, 66^c^, 66^c^ | 00 | 0/11 0% |
| Radiographic joint damage |  | 72 | 44^c^, 57 | 0 | 1/3 33% |
| Aerobic fitness | 62 |  | 40, 63 | 0 | 1/3 33% |
| Strength and muscle function | 51^b^ |  | 40, 41, 63, 64^ac^, 64^ac^ | 00 | 1/6 17% |
| Range of motion |  |  | 40, 41 | 0 | 0/2 0% |
| Balance |  |  | 40, 41, 64^ac^, 64^ac^ | 00 | 0/4 0% |
| Function (inability) |  | 35, 36, 44^c^, 46, 46^c^, 47^c^, 51^b^, 52^c^, 52^c^, 59^c^, 59^c^, 61^c^, 64^ac^, 65^ac^ | 34, 35, 39^a^, 40, 41, 42^c^, 43, 45, 49, 50, 55, 57, 57, 66^c^, 64^ac^, 69^c^ | ?? | 14/30 47% |
| Gait Speed |  |  | 64^ac^, 64^ac^ | 0 | 0/2 0% |
| Pain |  | 53, 61^c^ | 34, 35, 35, 36, 40, 41, 43, 45, 47^c^, 54^bc^, 55, 57, 62, 63, 66^c^ | 00 | 2/17 12% |
| Fatigue |  | 45, 51^b^, 55, 55, 63, 66^c^ | 34, 35, 35, 36, 46, 46^c^, 50, 55, 55, 55, 58, 62 | 00 | 6/18 33% |
| Sleep (good/high) | 51^b^, 59^c^ |  | 55, 59^c^, 66^c^ | ? | 2/5 40% |
| Stiffness |  | 34 |  | - | 1/1 100% |
| Other (physical) |  |  |  |  |  |
| Waist circumference |  | 70 | 39^a^, 47^c^ | 0 | 1/3 33% |
| Waist to hip ratio |  | 33 | 33, 46, 46^c^ | 0 | 1/4 25% |
| Abdominal obesity |  | 70 | 72 | ? | 1/2 50% |
| Fat Mass Index |  |  | 39^a^ | 0 | 0/1 0% |
| Lean/fat mass | 51^b^ |  |  | + | 1/1 100% |
| Body fat |  | 68 |  | - | 1/1 100% |
| Diabetes |  |  | 33, 55, 66^c^, 72 | 00 | 0/4 0% |
| Fat Free Mass Index |  |  | 39^a^ | 0 | 0/1 0% |
| Plasma glucose/insulin |  | 39^a^ |  | - | 1/1 100% |
| Total cholesterol |  |  | 33, 39^a^ | 0 | 0/2 0% |
| Cholesterol efflux capacity |  |  | 33 | 0 | 0/1 0% |
| Low-density lipoprotein |  |  | 33, 33, 33, 33, 39^a^, 52^c^, 52^c^ | 00 | 0/7 0% |
| High-density lipoprotein | 33, 33, 33, 33, 39^a^, 52^c^, 52^c^ |  | 33, 33 | ++ | 7/9 78% |
| Tryglycerides |  |  | 33, 52^c^, 52^c^, 72 | 00 | 0/4 0% |
| Dyslipidaemia |  |  | 72 | 0 | 0/1 0% |
| Apolipoprotein A1 | 39^a^ |  |  | + | 1/1 100% |
| Apolipoprotein B |  |  | 39^a^ | 0 | 0/1 0% |
| Oxidized LDL |  |  | 39^a^ | 0 | 0/1 0% |
| Antibodies against phosphorylcholine | 39^a^ |  |  | + | 1/1 100% |
| Anti-citrullinated protein antibodies |  |  | 34, 72 | 0 | 0/2 0% |
| Erythrocyte sedimentation rate |  |  | 34, 39^a^, 40, 46, 46^c^, 47^c^, 57, 61^c^, 63, 65^ac^, 66^c^ | 00 | 0/11 0% |
| C-reactive protein |  | 33, 61^c^ | 34, 40, 46, 46^c^, 51^b^, 59^c^, 59^c^, 63, 65^ac^, 66^c^, 72 | 00 | 2/13 15% |
| Matrix metalloproteinase (MMP-3) |  |  | 61^c^ | 0 | 0/1 0% |
| RF positive |  |  | 34, 66^c^ | 0 | 0/2 0% |
| Cardiovascular risk / disease |  | 46^c^ | 46, 46, 46^c^, 66^c^, 72 | 00 | 1/6 17% |
| Hypertension |  | 33 | 72 | ? | 1/2 50% |
| Systolic blood pressure |  | 52^c^, 52^c^ | 33 | - | 2/3 67% |
| Diastolic blood pressure |  | 52^c^, 52^c^ | 33 | - | 2/3 67% |
| Heart rate |  | 33, 51^b^ |  | - | 2/2 100% |
| Metabolic syndrome |  | 46 | 46^c^, 72 | 0 | 1/3 33% |
| Nutritional complications |  | 48^c^ | 48^c^ | ? | 1/2 50% |
| Augmentation Index |  |  | 33 | 0 | 0/1 0% |
| Pulse wave velocity |  | 33 | 33 | ? | 1/2 50% |
| Agatson Score |  |  | 33 | 0 | 0/1 0% |
| Insulin resistance |  | 33, 52^c^, 52^c^ |  | - | 3/3 100% |
| RA -related joint surgery |  | 50 |  | - | 1/1 100% |
| COPD |  |  | 66^c^ | 0 | 0/1 0% |
| Haemoglobin |  |  | 66^c^ | 0 | 0/1 0% |
| Psychological |  |  |  |  |  |
| Exercise beliefs and (outcome) expectations | 36, 38^c^, 54^bc^, 63 |  | 35, 35, 35, 35, 36, 43 | ?? | 4/10 40% |
| Motivation | 38^c^, 47^c^, 49, 54^bc^ |  | 47^c^, 53 | ++ | 4/6 67% |
| Subjective Vitality | 42^c^ |  |  | + | 1/1 100% |
| Self-efficacy | 35, 36, 43, 47^c^, 47^c^, 53 |  | 35, 50, 63, 66^c^ | ++ | 6/10 60% |
| Depression / anxiety |  | 42^c^, 51^b^ | 35, 35, 55, 62, 63, 66^c^ | 00 | 2/8 25% |
| Life worries |  |  | 38^c^ | 0 | 0/1 0% |
| Health perception (good global health / assessment) | 34, 50, 61^c^ |  | 34, 35, 35, 36, 40, 41, 50, 55, 57, 59^c^, 59^c^ | 00 | 3/14 21% |
| Fear Avoidance Beliefs |  | 37 | 35, 35, 36, 56 | 00 | 1/5 20% |
| Other (psychological) |  |  |  |  |  |
| Life beliefs |  |  | 63 | 0 | 0/1 0% |
| Health locus of control |  |  | 41, 56 | 0 | 0/2 0% |
| Pain and impairment relationship |  |  | 56 | 0 | 0/1 0% |
| Health-related quality of life | 53, 53, 65^ac^ |  | 46, 46, 46^c^, 46^c^, 50, 54^bc^, 65^ac^ | 00 | 3/10 30% |
| Individual SF-36 measures (health status) |  | 34, 34, 50 | 44^c^, 44^c^, 44^c^, 44^c^, 44^c^, 44^c^, 44^c^, 44^c^, 65^ac^, 65^ac^, 65^ac^, 65^ac^, 65^ac^, 65^ac^, 65^ac^, 65^ac^, 65^ac^, 66^c^, 66^c^, 66^c^ | 00 | 3/23 13% |
| Arthritis Impact |  |  | 34 | 0 | 0/1 0% |
| Beliefs about causes of fatigue |  |  | 66^c^, 66^c^ | 0 | 0/2 0% |
| Coping strategies (fatigue) |  |  | 66^c^, 66^c^, 66^c^, 66^c^, 66^c^, 66^c^ | 00 | 0/6 0% |
| Fatigue catastrophizing |  |  | 66^c^, 66^c^, 66^c^ | 0 | 0/3 0% |
| Social |  |  |  |  |  |
| Social support | 36, 42^c^ | 35 | 35, 35, 35, 43, 49, 63 | 00 | 2/9 22% |
| Other |  |  |  |  |  |
| Previous levels of PA | 35, 35, 41 |  |  | + | 3/3 100% |
| Medications / biologics |  | 46, 46^c^ | 39^a^, 39^a^, 39^a^, 47^c^, 47^c^, 47^c^, 47^c^, 50, 50, 50, 50, 50, 51^b^, 66^c^, 66^c^, 66^c^, 66^c^, 72, 72, 72 | 00 | 2/22 9% |
| Hospital admission /length |  | 60, 60 |  | - | 2/2 100% |
| Goal Achievement |  |  | 53 | 0 | 0/1 0% |

*Note.* ^a^ = female participants only. ^b^ = studies that investigated physical inactivity. ^c^ = associations from objective measures of PA. In the associations column the following applies: + = positive association; - = negative association; ? = indeterminant/inconsistent; 0 = no association. If four or more studies indicate the same association the codes are ++, - -, ?? and 00. The code is based on the percentage of studies supporting an association: 0-33% = 0; 34-59% = ?; and 60-100% = + or -.
