# Supplementary material for: Correlates of physical activity in adults with spondyloarthritis and rheumatoid arthritis: a systematic review
Source: Rheumatol Int. 2022 Jun 8;42(10):1693–713. doi: 10.1007/s00296-022-05142-z (PMC9439989; doi:10.1007/s00296-022-05142-z)
Supplement: Supplementary file 2 — Supplementary file2 (DOCX 23 KB) [file 296_2022_5142_MOESM2_ESM.docx]

Article title: Correlates of physical activity in adults with spondyloarthritis and rheumatoid arthritis: a systematic review

Journal: Rheumatology International

Thomas Ingram^1, 2*^, Raj Sengupta^2, 3^, Martyn Standage^1^, Rosie Barnett^1,2^ and Peter Rouse^1^

^1^ Department for Health, University of Bath, Bath, UK; ^2^ Royal National Hospital for Rheumatic Diseases, Royal United Hospitals NHS Foundation Trust, Bath, UK; ^3^ Department of Pharmacy & Pharmacology, University of Bath, Bath, UK

*Corresponding author: Thomas Ingram. tai23@bath.ac.uk

**Supplementary Table 2** Summary of study results based on the most adjusted or final model statistics for each variable - Spondyloarthritis

| Variable | Positive relationship | Negative relationship | No Relationship | Assoc. | % Studies |
| --- | --- | --- | --- | --- | --- |
| Sociodemographics |  |  |  |  |  |
| Age | 78, 83 | 78 | 74, 76, 82^c^, 83^c^ | 00 | 2/7 29% |
| Gender (female) | 78 | 78 | 74, 76, 82^c^, 82^c^ | 00 | 1/6 17% |
| Marital status (married) |  | 74 | 76 | ? | 1/2 50% |
| Employment (employed) |  | 76 | 74 | ? | 1/2 50% |
| Educational level |  |  | 76, 78, 78 | 0 | 0/3 0% |
| Raised children under 12 years |  |  | 76 | 0 | 0/1 0% |
| Smoking |  | 78 | 78 | ? | 1/2 50% |
| Physical |  |  |  |  |  |
| Comorbidities |  |  | 76 | 0 | 0/1 0% |
| Symptom duration | 74 |  | 76 | ? | 1/2 50% |
| Diagnosis duration |  | 82^c^ | 74, 82^c,^ 83, 83^c^ | 00 | 1/5 20% |
| Peripheral Joint involvement |  |  | 74 | 0 | 0/1 0% |
| Disease activity | 74, 82^c^ | 73, 73, 73, 73, 77, 78 | 73^c^, 73^c^, 75, 76, 76, 78, 80^c^, 80^c^, 82^c^, 83, 83^c^ | 00 | 6/19 32% |
| Erythrocyte sedimentation rate |  | 73^c^ | 73, 73 | 0 | 1/3 33% |
| C-reactive protein |  | 73^c^ | 73, 73, 76 | 0 | 1/4 25% |
| Function (inability) |  | 73, 73, 73^c^, 75, 78, 78, 82^c^, 83^c^ | 74, 76, 80^c^, 82^c^, 83 | - - | 8/13 62% |
| Spinal immobility |  |  | 74 | 0 | 0/1 0% |
| Occiput-to-wall distance |  | 73 | 73, 73^c^ | 0 | 1/3 33% |
| Chest expansion |  |  | 73, 73, 73^c^ | 0 | 0/3 0% |
| Modified Schober test | 73, 73^c^ |  | 73 | + | 2/3 67% |
| Lateral spinal flexion | 73^c^ |  | 73, 73 | 0 | 1/3 33% |
| Cervical rotation | 73, 73^c^ |  | 73 | + | 2/3 67% |
| Radiographic signs |  |  | 76 | 0 | 0/1 0% |
| Axial pain |  |  | 76 | 0 | 0/1 0% |
| Fatigue |  | 83, 83, 83 | 83, 83, 83^c^, 83^c^, 83^c^, 83^c^, 83^c^ | 00 | 3/10 30% |
| Psoriatic arthritis subtype |  | 78 | 78 | ? | 1/2 50% |
| Undifferentiated spondyloarthritis subtype |  |  | 78, 78 | 0 | 0/2 0% |
| Inflammatory bowel disease-related arthritis |  |  | 78, 78 | 0 | 0/2 0% |
| Uveitis |  |  | 76 | 0 | 0/1 0% |
| Psoriasis |  |  | 76 | 0 | 0/1 0% |
| Inflammatory bowel disease |  |  | 76 | 0 | 0/1 0% |
| HLA B27 antigen |  |  | 76 | 0 | 0/1 0% |
| Inflammatory back pain |  |  | 76 | 0 | 0/1 0% |
| Arthritis |  |  | 76 | 0 | 0/1 0% |
| Enthesitis |  |  | 76 | 0 | 0/1 0% |
| Dactylitis |  |  | 76 | 0 | 0/1 0% |
| Surgery due to axSpA |  |  | 76 | 0 | 0/1 0% |
| Body Mass Index (BMI) |  | 82^c^ | 76, 82^c^, 83, 83^c^ | 00 | 1/5 20% |
| Aerobic fitness (VO_2_max) | 80^c^ |  |  | + | 1/1 100% |
| Having Ankylosing spondylitis |  | 81 |  | - | 1/1 100% |
| Psychological |  |  |  |  |  |
| Health perceptions (overall / disease) |  |  | 74, 74, 76, 83, 83^c^ | 00 | 0/5 0% |
| Quality of life | 73, 73, 73^c^, 78, 78 |  | 80^c^ | ++ | 5/6 83% |
| Motivation | 75, 75, 75 | 75 | 75, 75 | ? | 3/6 50% |
| Perception of exercise |  |  | 76, 76 | 0 | 0/2 0% |
| Depression |  | 79 | 76, 78, 78 | 0 | 1/4 25% |
| Anxiety |  |  | 76, 78, 78, 79 | 00 | 0/4 0% |
| Environmental |  |  |  |  |  |
| Seasonal variation (summer) | 77 |  |  | + | 1/1 100% |
| Other |  |  |  |  |  |
| Previous levels of PA | 74 |  |  | + | 1/1 100% |
| Medication / biologics | 81 |  | 74, 76, 76 | 0 | 1/4 25% |
| Age of anti-TNF therapy |  | 81 |  | - | 1/1 100% |
| Physiotherapy |  |  | 76 | 0 | 0/1 0% |

*Note.*^c^ = Associations from objective measures of PA. In the associations column the following applies: + = positive association; - = negative association; ? = indeterminant/inconsistent; 0 = no association. If four or more studies indicate the same association the codes are ++, - -, ?? and 00. The code is based on the percentage of studies supporting an association: 0-33% = 0; 34-59% = ?; and 60-100% = + or -.
