# Supplementary material for: Correlates of physical activity in adults with spondyloarthritis and rheumatoid arthritis: a systematic review
Source: Rheumatol Int. 2022 Jun 8;42(10):1693–713. doi: 10.1007/s00296-022-05142-z (PMC9439989; doi:10.1007/s00296-022-05142-z)
Supplement: Supplementary file 3 — Supplementary file3 (DOCX 52 KB) [file 296_2022_5142_MOESM3_ESM.docx]

Article title: Correlates of physical activity in adults with spondyloarthritis and rheumatoid arthritis: a systematic review

Journal: Rheumatology International

Thomas Ingram^1, 2*^, Raj Sengupta^2, 3^, Martyn Standage^1^, Rosie Barnett^1,2^ and Peter Rouse^1^

^1^ Department for Health, University of Bath, Bath, UK; ^2^ Royal National Hospital for Rheumatic Diseases, Royal United Hospitals NHS Foundation Trust, Bath, UK; ^3^ Department of Pharmacy & Pharmacology, University of Bath, Bath, UK

*Corresponding author: Thomas Ingram. tai23@bath.ac.uk

**Supplementary Table 3** Summary of positive and negative statistics of variables related to PA – Rheumatoid arthritis

| **Citation** | **PA measure** | **Type of PA** | **Correlate (+/- only)** | **Stat Test** | **Statistic** |
| --- | --- | --- | --- | --- | --- |
| Byram et al. [33] | Customized interview / 2011 Compendium of PA | Exercisers vs non- exercisers  Exercisers vs non- exercisers  Exercisers vs non- exercisers  Exercisers vs non- exercisers  Exercisers vs non- exercisers  Duration (30 min increases)  Exercisers vs non- exercisers  Exercisers vs non- exercisers  Duration (30 min increases)  Exercisers vs non- exercisers  Duration (30 min increases)  Exercisers vs non- exercisers  Duration (30 min increases)  Exercisers vs non-exercisers | DAS28  CRP  Hypertension  Heart rate  Waist-hip ratio  Waist-hip ratio  HOMA (insulin resistance)  HDL particle concentration  HDL particle concentration  HDL small particle concentration  HDL small particle concentration  Pulse wave velocity  Pulse wave velocity  Augmentation Index | Mann-Whitney U or Chi - Square  Mann-Whitney U or Chi - Square  Mann-Whitney U or Chi - Square  Mann-Whitney U or Chi - Square  Mann-Whitney U or Chi - Square  Regression  Mann-Whitney U or Chi - Square  Regression  Regression  Regression  Regression  Regression  Regression  Regression | p = 0.02, p = 0.01*  p = 0.009, p = 0.03*  p = 0.03, p = 0.01*  p = 0.07, p = 0.05*  p = 0.03, p = 0.008*  Not sig.  p = 0.03, p = 0.05*  p = 0.04, p = 0.004*, p = 0.02*  beta/OR 0.509, 95%CI 0.088, 0.930, p = 0.019* & 0.024*  p = 0.005, p = 0.003*, p = 0.01*  beta/OR 0.550, 95%CI 0.183, 0.918, p = 0.004* & 0.006*  p = 0.008, p = 0.005*, p = 0.02  Not sig.  P = 0.05, p = 0.10*, p = 0.12* |
| Conigliaro et al. [34] | IPAQ | Physical exercise vs no physical exercise | DAS28  Stiffness, VAS  Global, VAS  SF-36 physical lim  SF-36 vitality | Mann-Whitney U and Chi-square | p = 0.004  p = 0.005  p = 0.003  p = 0.04  p = 0.01 |
| Demmelmaier et al. [35] | IPAQ  Exercise Stage Assessment Instrument (ESAI) for maintained HEPA  PA trajectories Stable high, decreasing, stable low | High vs dec  High vs dec  High vs low  High vs low  High vs low (MvsL)  High vs low (HvsL)  High vs Dec (MvsL)  High vs Dec  High vs Low | Age  Sex  Sex  Income  Disease duration  Comorbidities  Pain, VAS  Fatigue, VAS  Health, VAS  Anxiety / Depression  HAQ (act lim)  Self-efficacy for exercise (ESES)  Self-efficacy for exercise (ESES)  Social support for exercise, fa (SSEB)  Social support for exercise, fr (SSEB)  Fear-avoidance beliefs (mFABQ)  Outcome expectations on health, NRS  Outcome expectations on RA, NRS  Maintained HEPA  Maintained HEPA | Chi square or unpaired t-test (differences between all groups) / Multinomial logistic regression | p = 0.0007 / OR 0.34, 95%CI 0.14-0.81, p = 0.015  p = 0.0001 / OR 0.7, 95%CI 0.51-0.98, p = 0.036  p = 0.0001 / OR 0.5, 95% CI 0.37 – 0.67, p = <0.0001  p = 0.0006 / not sig  p = 0.0001 / not sig  p = 0.026 / not sig  p = 0.004 / not sig  p = 0.047 / not sig  p = 0.001 / not sig  p = 0.003 / not sig  p = 0.0001 / OR 0.58, 95%CI 0.34-0.96, p = 0.035  p = 0.0001 / OR 1.49, 95%CI 1.04-2.15, p = 0.030  p = 0.0001 / OR 1.77, 95%CI 1.22-2.56, p = 0.003  p = 0.003 / not sig  p = 0.005 / OR 0.61, 95%CI 0.37-0.99, p = 0.047  p < 0.0001 for mean, p = 0.001 for below median difference / not sig  p = 0.0001 for mean, p = 0.002 for <10 / not sig  p = 0.0001 / not sig  p < 0.0001 / OR 1.53, 95%CI 1.03-2.25, p = 0.034  p < 0.0001/ OR 2.9, 95%CI 2.03-4.15, p < 0.0001 |
| Demmelmaier et al. [36] | IPAQ-SF  Exercise Stage Assessment Instrument (ESAI) for maintained HEPA | Current HEPA  Maintained HEPA  Maintained HEPA  Current HEPA  Maintained HEPA  Current HEPA  Maintained HEPA  Current HEPA  Current HEPA  Current HEPA  Current HEPA  Maintained HEPA  Current HEPA  Maintained HEPA  Current HEPA  Maintained HEPA  Current HEPA  Maintained HEPA  Current HEPA  Maintained HEPA  Current HEPA  Maintained HEPA  Current HEPA  Maintained HEPA  Current HEPA  Maintained HEPA  Current HEPA  Maintained HEPA  Current HEPA  Maintained HEPA | Age (lower ages)  Sex  Education (higher)  Income  Adults in home (3/4)  Child <18yrs (0)  Language comprehension  Comorbidities (0)  Pain, VAS (low)  Fatigue, VAS (low)  HAQ-DI (low)  Self-efficacy for exercise (ESES) high  Outcome expectations on health (med or high)  Outcome expectations on RA, NRS (med or high)  Health, VAS (good)  Fear- avoidance (mFABQ) (low)  Social support for exercise (SSES) Mod | Logistic regression (univariate / adj. & multiple imputation) | OR 1.23, 95%CI 1.03-1.48 / OR 0.57, 95%CI 0.37-0.90*  OR 2.24, 95%CI 1.39-3.61 / OR 1.91, 95%CI 1.10-3.32*  Not sig / OR 1.38, 95%CI 1.03-1.85*  OR 2.26, 95%CI 1.86-2.75 / OR 1.57, 95%CI 1.24-1.99*  OR 1.60, 95%CI 1.19-2.16 / not sig*  OR 1.74, 95%CI 1.49-2.04 / not sig*  Not sig / OR 0.68, 95%CI 0.51-0.91*  OR 1.40, 95%CI 1.06-1.84 / not sig*  OR 0.67, 95%CI 0.49-0.89 / not sig*  OR 3.96, 95%CI 2.37-6.62 / OR 2.40, 95%CI 1.38-4.19*  OR 2.10, 95%CI 1.66-2.66 / not sig*  OR 1.48, 95%CI 1.20-1.83 / not sig*  OR 2.29, 95%CI 1.91-2.76 / not sig*  OR 1.59, 95%CI 1.18-2.14 / not sig*  OR 2.26, 95%CI 1.87-2.74 / not sig*  OR 2.23, 95%CI 1.65-3.01 / OR 1.73, 95%CI 1.12-2.67*  OR 4.09, 95%CI 3.24-5.16 / OR 2.32, 95%CI 1.70-3.17*  OR 2.82, 95%CI 1.96-4.06 / not sig*  OR 1.76, 95%CI 1.45-2.14 / OR 1.47, 95%CI 1.20-1.81*  OR 2.13, 95%CI 1.47-3.09 / OR 1.81, 95%CI 1.24-2.66  OR 2.30, 95%CI 1.96-2.69 / not sig*  OR 3.19, 95%CI 2.37-4.29 / OR 1.70, 95%CI 1.19-2.44*  OR 2.62, 95%CI 2.24-3.05 / OR 1.58, 95%CI 1.30-1.93*  OR 3.27, 95%CI 2.47-4.33 / OR 1.66, 95%CI 1.19-2.34*  OR 2.78, 95%CI 2.28-3.39 / not sig*  OR 1.93, 95%CI 1.45-2.58 / not sig*  OR 2.04, 95%CI 1.74-2.39 / not sig*  OR 2.00, 95%CI 1.58-2.54 / not sig*  OR 2.33, 95%CI 1.72-3.16 / OR 1.55, 95%CI 1.13-2.13*  OR 2.34, 95%CI 1.71-3.20 / OR 1.46, 95%CI 1.03-2.07* |
| Demmelmaier et al. [37] | IPAQ  ESAI | Current HEPA  Maintained HEPA | Fear-avoidance beliefs, trajectories (mFABQ) | Chi-square or unpaired t-test | p < 0.0001  p < 0.0001 |
| Ehrlich-Jones et al. [38] | GT1M Actigraph | Average daily accelerometer counts | Increased PA beliefs (customized scale)  Increased motivation for PA (customized based on perceived competence scale) | Multiple linear regression | p = 0.001 / p = 0.032*  p = 0.003 / p = 0.007* |
| Elkan et al. [39] | IPAQ | MET hours 1^st^ quartile vs 4^th^ quartile (total PA) | Higher plasma glucose/insulin  Lower HDL  Lower Apolipoprotein (apoA1)  Lower anti-bodies against phosphorylcholine (anti-PC) | Spearman rank correlation | p = 0.05  p = 0.05 or p = 0.004  p = 0.005  P = 0.016 |
| Eurenius et al. [40] | Customized self-report | Levels of PA | Age  Sex | Mann-Whitney U test and Spearman’s rank order correlation | Women >65 yrs less active than younger women or men  Women >65 yrs less active than younger women or men  (r and p not reported) |
| Eurenius et al. [41] | Customized self-report | High PA | Previous PA (high PA at baseline) | Simple Logistic regression and multiple logistic regression (both) | OR 3.85, 95%CI 1.67-9.09, p = 0.001 |
| Fenton et al. [42] | GT3X Accelerometer (actigraph) - LPA | Light Physical Activity | Depression (HADS)  Important other autonomy support (IOCQ)  Subject vitality (SVS) | Bivariate Pearson’s correlation / Path analysis | r = -0.29, p < 0.05  r = 0.37, p < 0.01 / (ꭓ2 (2) = 2.44, p = 0.30, CFI = 0.99, SRMR = 0.05, RMSEA = 0.07)  r = 0.27, p < 0.05 |
| Greene et al. [43] | Physical activity and disability survey (PADS) | Total PA time | Arthritis Self-Efficacy Scale (ASES) | Multivariate Regression | R^2^ = .12; p = 0.01* |
| Hashimoto et al. [44] | Actigraph mini-motionlogger, omnidirectional accelerometer | Mean Activity Count (MAC) | Activity limitation (HAQ-DI) | Spearman’s correlation | r = -0.52, p < 0.05 |
| Henchoz et al. [45] | PA Frequency Questionnaire (PAFQ) | Active vs sedentary (t-test)  TEE (regression) | Age  HAQ  DAS28  Pain, VAS  Fatigue, VAS | t-test /  Pearson’s & simple linear regression / multiple linear regression | r = -0.20, p < 0.001 / p = 0.027*  p = 0.010 / not sig / not sig*  p = 0.013 / r = -0.22, p = 0.032 / not sig*  p = 0.004 / not sig / not sig*  p = 0.023 / r = -0.22, p = 0.029 / p = 0.028* |
| Hernández-Hernández et al. [46] | IPAQ and RT3 triaxial accelerometer | MVPA  MVPA  MET-min/week  MET-min/week  Total VM  Total VM  MET-min/week  Total Kcal/day  MVPA  MET-min/week  Total VM  MVPA  (Met-min/week and total VM used) | High DAS28-ESR  High DAS28-CRP  High HAQ-DI  Framlingham / ATP III) – Cardio risk  Metabolic syndrome  Corticosteroid intake | Univariate reg  / Multivariate analysis* | r = -0.42, p = 0.02 / not sig*  r = -0.41, p = 0.049 / not sig*  B (95% CI) -544 (-1082, 5) p = 0.04*  B (95% CI) -1623 (-2742, -503) p = 0.00*  B (95% CI) -43 (-81, -6.46) p = 0.02*  B (95%CI) -10.69 (-21.02, -0.34), p = 0.04*  B (95% CI) -93.9 (-153.1, -34.7) p = 0.00*  B (95% CI) -219 (-407, -33) p = 0.02*  B (95% CI) -83.17 (-153.57, 12.77) p = 0.02*  B (95% CI) -1842 (-2823, -860) p = 0.00*  B (95% CI) -46.42 (-68.41, -24.42) p = 0.00*  B (95% CI) -56.02 (-109.98, -2.05) p = 0.04* |
| Huffman et al. [47] | RT3 triaxial accelerometer | Time spent in exercise | Gender (F)  Comorbidity index  High DAS28-ESR  HAQ-DI  End PA motivation  End PA self-efficacy  Strength PA self-efficacy | Spearman correlation | r = -0.39, p < 0.05  r = -0.40, p < 0.05  r = -0.31, p < 0.05  r = -0.32, p < 0.05  r = 0.34, p < 0.05  r = 0.42, p < 0.05  r = 0.36, p < 0.05 |
| Hugo et al. [48] | Sensewear armband accelerometer / REE measured using a Sensormedics VMX29N apparatus | PA duration  Levels of PA (METs)  PA duration  Levels of PA (METs)  Levels of PA (METs) | Disease duration  Nutritional complications (RC or MS) | Regression & Binary logistic regression  Uni / Multivariate logistic regression | b = -0.29, p = 0.043 / b = -0.23, p = 0.09* (not sig)  b = -0.31, p = 0.03 / b = -0.24, p = 0.08* (not sig)  b = -0.31, p = 0.027 / p = 0.09* (not sig)  b = -0.37, p = 0.008 / p = 0.03*  exp(B) = 0.04, p = 0.016, 95%CI 0.003, 0.551 / exp(B) = 0.03, p = 0.009, 95%CI 0.002, 0.422 (PA not DV) |
| Hurkmans et al. [49] | Short questionnaire to assess HEPA  (SQUASH) | Total PA (mins) | Age  Gender (F)  Educational level  Disease duration  Disease activity (RADAI)  Treatment Self-Regulation Questionnaire | Pearson Correlation & Multiple hierarchical regression | r = -0.493, p= 0.01 / beta (β) -33.47 (-0.36), SE B 7.78, p = 0.000; R2 = 0.255, F for change R2 = 7.291, p<0.001*  r = 0.130, p = 0.05 / not sig*  r not reported, p = 0.016 / not sig*  r = -0.192, p = 0.002 / not sig*  r = -0.159, p = 0.015 / not sig*  r = 0.299, p = 0.01 / beta (β) 138.70 (0.17), SE B 69.88, p = 0.049; R2 = 0.255, F for change R2 = 7.291, p < 0.001 |
| Iverson et al. [50] | Nurses Health Study II Physical Activity Questionnaire (NHSPAQ II) | Meeting ODPHP MVPA recommendations | Age  Ethnicity (caucasian)  Educational level  Employment status  Marital status  Any alcohol use  BMI  Patient global assessment of function  Physician global assessment (rheumatoid arthritis severity scale)  Physical function (MDHAQ)  Fatigue, VAS (CLINHAQII)  RA related joint surgery  Self-efficacy  Mental health disorder (MHI-5)  QoL (EQ-5D) | Repeated measures logistic regression* / Multivariable repeated measures logistic model* | ≥ 69yrs OR= 0.52, 95%CI 0.36-0.74; ≥ 62-69yrs OR= 0.68, 95%CI 0.48-0.97; ≥53-62 yrs OR= 0.65, 95%CI 0.46-0.92* / (≥69yrs OR= 0.58, 95%CI 0.36-0.92*  Caucasian OR= 2.85, 95%CI 1.39-5.81* / OR= 2.95, 95%CI 1.29-6.75*  college grad OR= 1.81, 95%CI 1.33-2.46* / not sig*  Full time employment OR= 1.36, 95%CI 1.08-1.75* / not sig*  Married or living with significant other OR= 1.57, 95%CI 1.17-2.09* / not sig*  OR= 1.46, 95%CI 1.17-1.81 / not sig*  Obese Class III OR= 0.18, 95%CI 0.06-0.49; Obese Class I/II OR= 0.52, 95%CI 0.37-0.72; Overweight OR= 0.61, 95%CI 0.46-0.81* / Obese Class III OR= 0.24, 95%CI 0.08-0.74; Obese Class I/II OR 0.60, 95%CI 0.41-0.88; Overweight OR 0.69, 95%CI 0.50-0.95*  ≥50 OR= 0.37, 95%CI 0.26-0.52; ≥20-50 OR= 0.49, 95%CI 0.35-0.68; ≥10-20 OR= 0.55, 95%CI 0.40-0.76* / ≥10-20 OR= 0.57, 95%CI 0.39-0.83*  >3 OR= 0.71, 95%CI 0.52-0.97* / not sig*  OR= 0.38, 95%CI 0.27-0.53* / OR= 0.59, 95%CI 0.34-1.01* (BL)  (≥ 70 OR= 0.51, 95%CI 0.36-0.74; ≥ 40-70 OR= 0.60, 95%CI 0.43-0.83; ≥15-40 OR= 0.70, 95%CI 0.51-0.95* / not sig*  OR= 0.71, 95%CI 0.52-0.97*  ≥ 80 OR= 2.18, 95%CI 1.27-3.76* / not sig*  OR= 0.49, 95%CI 0.35-0.68* / OR= 0.63, 95%CI 0.41-0.95*  OR= 5.49, 95%CI 2.34-12.89* / not sig* |
| Katz et al. [51] | IPAQ | Physical inactivity | Current smoking  Obesity  Disease activity (RADAI)  Cardiorespiratory fitness (heart rate)  Muscle weakness (hip strength)  HAQ  Lean/fat mass ratio  Sleep quality (PSQI)  Depressive symptoms (PHQ)  Fatigue (FSI) | Spearman correlation  Bivariate analysis / Multivariate linear regression* | r = 0.22, p < .01  r = 0.29, p < .0001  r = 0.23, p < .01  r = 0.23, p < .01  r = -0.22, p < .05  r = 0.28, p < .001  r = -0.22, p < .01  r = 0.18, p < .05  r = 0.24, p < .001  B 1.17, p = .0002 / not sig multivariate, but sig in mediation (fatigue DV) |
| Khoja et al. [52] | Sensewear armand biaxial accelerometer, heat flux, skin temp and galvanic skin response | Very Light PA  Light PA  Moderate PA  Very Light PA  Light PA  Very Light PA  Light PA  Moderate PA  Very Light PA  Light PA  Moderate PA  Light PA  Very Light PA  Light PA  Moderate PA  Very Light PA  Light PA  Moderate PA  Very Light PA  Light PA  Moderate PA | BMI  DAS28  HAQ  HDL  Triglycerides  Diastolic blood pressure  Systolic blood pressure  Insulin resistance (HOMA-IR) | Spearman’s correlation / Linear regression model*  PA not DV in regression | r = -0.651, p < 0.05 / B = -.65, 95%CI (-.75, -.51), R2Δ .40, p <.001*  r = -0.790, p < 0.05 / B = -.74, 95%CI (-.81, -.63), R2Δ .52, p <.001*  r = -0.426, p < 0.05 / B = -.45, 95%CI (-.60, -.28), R2Δ .19, p <.001*  r = -0.274, p < 0.05 / B = -.20, 95%CI (-.35, -.02), R2Δ .04, p =.040*  r = -0.280, p < 0.05 / not sig*  r = -0.277, p < 0.05 / B = -.23, 95%CI (-.38, -.09), R2Δ .05, p =.027*  r = -0.261, p < 0.05 / B = -.23, 95%CI (-.38, -.09), R2Δ .05, p =.026*  r = -0.384, p < 0.05 / B = -.34, 95%CI (-.47, -.20), R2Δ .11, p =.001*  r = 0.246, p < 0.05 / B = .21, 95%CI (.01, .39), R2Δ .04, p =.021*  r = 0.249, p < 0.05 / B = .30, 95%CI (.11, .47), R2Δ .08, p =.001*  not sig / B = .19, 95%CI (.01, .37), R2Δ .03, p =.044*  r = -0.202, p < 0.05 / not sig*  r = -0.373, p < 0.05 / B = -.40, 95%CI (-.55, -.22), R2Δ .15, p <.001*  r = -0.239, p < 0.05 / B = -.25, 95%CI (-.43, -.05), R2Δ .06, p =.010*  r = -0.223, p < 0.05 / B = -.21, 95%CI (-.39, -.01), R2Δ .04, p =.029*  r = -0.379, p < 0.05 / B = -.35, 95%CI (-.51, -.16), R2Δ .12, p <.001*  r = -0.200, p < 0.05 / B = -.28, 95%CI (-.45, -.09), R2Δ .07, p =.006*  r = -0.328, p < 0.05 / B = -.28, 95%CI (-.45, -.09), R2Δ .08, p =.006*  r = -0.348, p < 0.05 / B = -.32, 95%CI (-.45, -.12), R2Δ .09, p =.002*  r = -0.507, p < 0.05 / B = -.43, 95%CI (-.57, -.25), R2Δ .17, p <.001*  r = -0.364, p < 0.05 / B = -.29, 95%CI (-.46, -.10), R2Δ .08, p =.004* |
| Knittle et al. [53] | The short questionnaire to assess HEPA (SQUASH) | PA score | T1 age  T2 arthritis pain (3 items on RADAI)  T1 self-efficacy (self-regulation skills battery)  T1 regulation style (TSRQ)  T2 physical QoL (SF-36 Dutch)  T2 mental QoL (SF-36 Dutch) | Pearson’s correlation (bivariate) / mediation analysis (a path) controlling for other variables* | r = -0.54, p ≤ 0.01  r = -0.26, p ≤ 0.01  r = 0.25, p ≤ 0.01 / p < 0.05  r = 0.36, p ≤ 0.01 / not sig*  r = 0.43, p ≤ 0.01  r = 0.36, p ≤ 0.01 |
| Lee et al. [54] | GT1M Actigraph  (inactivity) | Inactive vs active  Inactive vs active  Physical inactivity  Physical inactivity  Physical inactivity | Age  Ethnicity  Weight (obese)  Lack of strong belief – customized  Lack of strong motivation – customized | Descriptive stats  Descriptive stats  Uni / multivariate logistic regression* | p < 0.05  p < 0.01  OR=2.14, 95% CI: 1.12, 4.10 / not sig*  OR=2.13, 95% CI:1.07, 4.26 / OR=2.47, 95% CI: 1.10, 5.56; AF=49.2%, 95% CI: 7.0, 76.4*  OR=2.24, 95%CI: 1.18,4.25 / OR=2.85, 95% CI: 1.31, 6.20; adjusted AF=53.1%, 95% CI: 21.7, 74.6* |
| Løppenthin et al. [55] | Leisure time physical activity questionnaire (Saltin & Grimby) and Physical Activity Scale (PAS) | Regular PA | Sex (male)  Age  DAS28  HAQ  Pain, VAS  Fatigue, MFI mental  Fatigue, MRI activity  Fatigue, MRI physical  Fatigue, MRI general  Global, VAS | Univariate logistic regression / elastic net procedure for building multivariate prediction models (2,500 bootstrap samples)* | OR 2.00, 95%CI 1.07-3.76, p = 0.02 / 51% selected only*  OR 1.03, 95%CI 1.00-1.05, p = 0.006 / 79% selected only*  OR 0.53, 95%CI 0.39-0.72, p < 0.001 / 82.0% selected, median OR 0.90 (2.5-97.5% quantile: 0.67-1.00)*  OR 0.36, 95%CI 0.22-0.72, p < 0.001 / 51% selected only*  OR 0.97, 95%CI 0.96-0.99, p = 0.0007 / 14% selected only*  OR 0.91, 95%CI 0.85-0.98, p = 0.01 / 11% selected only*  OR 0.68, 95%CI 0.59-0.78, p <0.001 / 97.5% selected, median OR 0.89 (2.5-97.5% quantile: 0.78-1.00)*  OR 0.81, 95%CI 0.75-0.87, p < 0.001 / 99.7% selected, median OR 0.91 (2.5-97.5% quantile: 0.82-0.97)*  OR 0.90, 95%CI 0.85-0.96, p = 0.001 / 29% selected only*  OR 0.98, 95%CI 0.97-0.99, p = 0.001 / 16% selected only* |
| Lundgren et al. [56] | Physical Activity Index (PAI) | Physical activity (quartiles) | Fear-avoidance beliefs (mFABQ)  Pain and impairment relationship (PAIRS)  Health locus of control (MHLC-C – internal, chance, doctors or odds) | Logistic regression | No significant associations |
| Malm et al. [57] | Three questions measuring self-reported health-enhancing PA over survey duration | Meeting WHO rec  Meeting WHO VI-PA rec  Meeting WHO MI-PA rec  Meeting WHO VI-PA rec | Sex (female)  Swollen Joint (from DAS28)  HAQ | Descriptive stats  Multiple logistic regression* | p = 0.01  p = 0.02  OR 0.98, 95%CI 0.957-0.998, p = 0.03 (study reported not predictive of meeting WHO MI-PA rec)  OR 1.28, 95%CI 1.018-1.609, p = 0.04 (study reported not predictive of meeting WHO VI-PA rec) |
| Mancuso et al. [58] | Paffenbarger Physical Activity and Exercise Index (PAEI) | Less physical activity | Fatigue (fatigue severity scale – FSS) | Bivariate analysis / multivariate model | 0.21, p = 0.02 / not sig in multivariate model with fatigue as DV |
| McKenna et al. [59] | The SWA multisensor PA monitor (accelerometry, skin temp, galvanic skin response, heat flux, near-body temp) | MVPA duration  TEE  PAEE  MVPA duration  TEE  PAEE  MVPA duration | Gender  DAS28  AvHAQ  Total Sleep time | 2-sample t-test  Pearson’s correlation | p <0.001, effect size -1.56  r = -0.371, p = 0.037  r = -0.443, p = 0.011  r = -0.454, p = 0.009  r = -0.411, p = 0.019  r = -0.503, p = 0.003  r = 0.415, p = 0.018 |
| Metsios et al. [60] | IPAQ | MET-mins/week  Overall PA  Vigorous PA  Overall PA  Vigorous PA  Achieving PA rec | Number of hospital admissions  Length of hospital admissions  Younger age  Lower CRP  Less swollen joints  Lower HAQ | Spearman’s rank correlation  Logistic regression*  Paired t-test or Mann-Whitney U test | rho = -0.262, p < 0.001  rho = -0.270, p < 0.001  rho = -0.231, p < 0.001  rho = -0.295, p < 0.001  exp(B) = 0.55, p = 0.02*  exp(B) = 0.55, p = 0.02*  p = 0.02  p = 0.001 |
| Mochizuki et al. [61] | Liferecorder - measures amount of PA by step count, PA intensity, moving distance and PA time. | With vs without PA achievement goal / Achievement of PA goal (≥2000 kcal / week or 286 kcal / day) – with vs without | Age  Disease duration  BMI  DAS28-CRP  CRP  HAQ  Pain, VAS  Global, VAS | Wilcoxon rank sum / Multivariate logistic regression* | p = 0.0012 / OR 0.926, 95%CI 0.882-0.966, p = 0.001*  p = 0.0039 / not sig*  p = 0.0046 / OR 1.180, 95%CI 1.039-1.357, p = 0.014*  p < 0.0001 / not sig*  p = 0.0312  p < 0.0001 / OR 0.229, 95%CI 0.046-0.819, p = 0.043*  p < 0.0001 / OR 0.969, 95%CI 0.941-0.994, p = 0.023*  p < 0.0001 |
| Munsterman et al. [62] | SQUASH | PA level  METhours/week | V0_2MAX_  Gender | Intercorrelations (from multiple regression) | 0.327, p ≤ 0.05  *p* = 0.01 |
| Neuberger et al. [63] | 1.) Mean minutes of aerobic exercise / week + mean minutes of aerobic portion of intervention (self-report). 2.) Mean minutes of aerobic exercise / week (self-report) | Minutes of exercise | Higher Global Fatigue Index of Multidimensional  Assessment Score  Exercise Benefits / Barriers Scale | CART analysis | Global fatigues scores ≥31 exercised 62 mins / week on average. Global fatigue score <31 exercised 85 mins / week on average.  In those with low fatigue: Those with scores of <117 exercised 62 mins / week on average. Those with scores of ≥117 exercised 95 mins / week on average. |
| Piva et al. [64] | SenseWear Professional v 6.1 activity monitor (accel, heat flux, skin temp, galvanic signal data | PAEE  # of steps  PAEE  # of steps  PAEE  # of steps  PAEE  # of steps  PAEE  # of steps  PAEE  # of steps  PAEE  # of steps  PAEE  # of steps | Age  Education  RA Duration  Comorbidities  5-Chair rise  Simple leg stance  HAQ  Gait speed | Pearson correlation (bivariate)  Multiple linear regression (zero order / semi partial correlation adjusted*) | r = -0.238, p < 0.05  r = -0.250, p < 0.05  r = 0.266, p < 0.05  r = 0.312, p < 0.05  not sig  r = -0 .299, p < 0.05  not sig  r = -0 .277, p < 0.05  not sig / not sig*  = -0 .479, p = 0.001 / not sig*  = 0.308, p = 0.035 / not sig*  not sig / not sig*  = -0 .376, p = 0.009 / not sig*  = -0.478, p = 0.001 / -0.277, P = 0.041*  = 0.365, p = 0.012 / not sig*  = 0.386, p = 0.007 / not sig* |
| Prioreschi et al. [65] | Actical acelerometer | Average activity counts / day  More PA vs less PA (median average activity counts) | Age  Disease duration  BMI  HAQ-DI  SF-36 (composite physical health)  SF-36 (composite physical health)  SF-36 (vitality)  SF-36 (composite mental health)  SF-36 (total) | Spearman’s or Pearson’s correlation  Student’s t-test | r = -0.441, p = 0.005  r = -0.406, p = 0.010  r = -0.361, p = 0.023  r = -0.343, p = 0.026 / r^2^ = 0.117, P = 0.026*  r = 0.326, p = 0.043  p = 0.05  p = 0.034  p = 0.05  p = 0.03 |
| Rongen-van Dartel et al. [66] | Ankle-worn actometer (Actilog version 4.1) | Low activity level vs high activity level  Daily activity (continuous)  Low vs high activity | Sex (female)  BMI  CIS fatigue  CIS fatigue | Student’s t t-test, Mann-Whitney U test or Chi-square linear regression model of fatigue (DV)* | p = 0.004  p = 0.004  p = 0.03  B = -0.082, 95%CI -0.14- -0.021, p = 0.008*  B = -4.42, 95%CI -8.059 - -0.78, p = 0.018* |
| Semanik et al. [67] | Yale PA survey  (& activity dimension summary index, ASDI) | ADSI score | Age  Employment status | Pearson’s correlation  ANOVA | r = -0.195, p < 0.01  F = 7.81, df = 2, p = 0.001 |
| Stavropoulos-Kalinoglou et al. [68] | IPAQ | PA | BMI  Body fat | Spearman’s correlation | r = -0.511, p = 0.000  r = -0.575, p = 0.000 |
| Tierney et al. [69] | Sensewear armband | TEE  PAEE  TEE  PAEE  TEE  PAEE  TEE  PAEE  TEE  PAEE  TEE  PAEE  TEE  PAEE | Age  Gender (female)  Employment  Smoking status  BMI  Disease activity (DAS28)  Activity limitation (HAQ-DI) | Pearson’s correlation and partial correlations* / multiple linear regression* | r = -0.507, p<0.001 / B -22.685, SE 3.743, p < 0.001, individual contribution to R^2^ 0.211, adjusted r^2^ for model = 0.667*  r = -0.344, p = 0.008 / B -7.796, SE 3.321, p = 0.023, individual contribution to R^2^ 0.038, Adjusted R^2^ for model = 0.604*  r = -0.615, p<0.001 / B -620.030, SE 91.074, p < 0.001, individual contribution to R^2^ 0.266, adjusted R^2^ for model = 0.667*  r = -0.622, p < 0.001 / B -450.975, SE 75.643, p < 0.001, individual contribution to R^2^ 0.243, Adjusted R^2^ for mode = 0.604*  r = 0.484, p<0.001 / r = 0.312, p = 0.018* / not sig*  r = 0.540, p < 0.001 / r = 0.447, p = 0.001* / B 271.794, SE 84.360, p = 0.002, individual contribution to R^2^ 0.071, adjusted R^2^ for model =0.604  not sig / r = -0.348, p = 0.008* / B -275.404, SE 98.375, p = 0.007, individual contribution to R^2^ 0.045, adjusted R^2^ for model = 0.667  r = -0.330, p = 0.011 / r = -0.411, p = 0.002* / B -233.149, SE 82.486, p = 0.007, individual contribution to R^2^ 0.055, Adjusted R^2^ for model = 0.604  r = 0.340, p=0.008 / r = 0.376, p = 0.004* / B 25.366, SE 8.320, p = 0.004, individual contribution to R^2^ 0.053, adjusted R^2^ for model = 0.667  not sig / not sig* / not sig*  r = -0.360, p=0.005 / not sig* / not sig*  r = -0.431, p = 0.001 / r = -0.304, p = 0.021* / not sig*  r = -0.325, p=0.012 / not sig* / not sig*  r = -0.395, p = 0.002 / r = -0.267, p = 0.045* / not sig* |
| Uutela et al. [70] | The Frequency Intensity Time (FIT) Index of Kasari | PA level (mean) | Presence of abdominal obesity  Waist circumference | T-test, bootstrapped-type t-test, permutation test, Wilcoxon test or χ2  Partial correlation | p < 0.001  > -0.3 (moderate)* |
| Van den Berg et al. [71] | Customized measure of PA (self-report) | Active vs inactive | Age  Gender (female)  Education | Independent samples t-test, Fisher’s Exact or Pearson Chi-square | p < 0.001  p = 0.04  p = 0.02 |
| Van der Goes et al. [72] | SQUASH | PA score | Age  Disease duration  BMI  DAS28  Sharp van der Heijde score – radiographic joint damage  Framlingham score  Cumulative GC dose  Abdominal obesity  Hypertension  Diabetes  Triglycerides  Metabolic syndrome  CVD  Glucocorticoid therapy  Cumulative GC dose | Spearman’s correlation / Multivariate linear regression*  t-test, Mann-Whitney U test or Chi-square | R^2^ = 0.247 for model, absolute beta -76, 95%CI -117 to -36, p < 0.001, standardised beta -0.272*  R= -0.164, p<0.05 / not sig*  R^2^ = 0.247, absolute beta -142, 95%CI -237 to -46, p < 0.01, standardised beta -0.221*  R = -0.316, p < 0.001 / R^2^ = 0.247, absolute beta -610, 95%CI -1011 to -208, p < 0.01, standardised beta -0.226*  R = -0.158, p < 0.05 / R^2^ = 0.247, absolute beta -6, 95%CI -11 to -1, p = 0.02, standardised beta -0.175*  R = -0.282, p = 0.001 / not sig*  R = -0.194, p < 0.02 / not sig*  P not reported / not sig*  P not reported / not sig*  P not reported / not sig*  P not reported / not sig*  P not reported / not sig*  P not reported / not sig*  P < 0.01 / not sig*  R = -0.194, *p* = 0.02 / not sig* |

*Note*. Based on the review criteria and process, this table contains associations related to PA parameters that may not be have been included within the associations table.

**Supplementary Table 4** Summary of positive and negative statistics of variables related to PA – Spondyloarthritis

| **Citation** | **PA measure** | **Type of PA** | **Correlates (+/- only)** | **Stat test** | **Statistic** |
| --- | --- | --- | --- | --- | --- |
| Arends et al. 2013 [73] | IPAQ, SQUASH and ActiGraph (GT1M) | IPAQ  SQUASH  IPAQ  SQUASH  Accelerometer  Accelerometer  Accelerometer  IPAQ  SQUASH  Accelerometer  SQUASH  SQUASH  Accelerometer  Accelerometer  SQUASH  Accelerometer  IPAQ  SQUASH  Accelerometer | BASDAI  ASDAS-CRP  ESR  CRP  BASFI  Occiput-to-wall distance (cm)  Modified Schober test (cm)  Lateral spinal flexion (cm)  Cervical rotation (degrees)  ASQoL | Spearman’s correlation / multivariate linear regression* | r = -0.220, p < 0.05 / sig*  r = -0.326, p < 001 / sig*  r = -0.243, p < 0.05 / sig*  r = -0.311, p < 0.01 / sig*  r = -0.283, p < 0.05 / not sig*  r = -0.460, p < 0.001 / sig*  r = -0.289, p < 0.05 / sig*  r = -0.387, p <0.001 / sig*  r = -0.476, p < 0.001 / sig*  r = -0.274, p < 0.05 / sig*  r = -0.297, p < 0.01 / sig*  r = 0.260, p < 0.05 / sig*  r = 0.338, p < 0.05 / sig*  r = 0.369, p <0.05 / sig*  r = 0.306, p < 0.01 / sig*  r = 0.320, p < 0.01 / sig*  r = -0.282, p <0.01 / sig*  r = -0.500, p < 0.001 / sig*  r = -0.356, p < 0.01 / sig* |
| Brodin et al. [74] | Customized scale | Exercise ≥2/week | Living alone (vs married or partner)  Symptom duration <26 yrs (vs ≥26 yrs)  Disease duration <14 yrs (vs ≥14 yrs)  BASDAI <4.05 (vs ≥4.05) / ≥4.05 (vs <4.05)  BASFI <3.80 (vs ≥3.80)  BAS G1 <4.20 (vs ≥4.20)  Previous exercise ≥2/week (vs irregular or ≤2/week | Simple logistic regression / multiple logistic regression | OR 3.97, 95%CI 1.07-14.71, p = 0.039 / OR 7.02, 95%CI 1.00-49.38, p = 0.050  Not sig / OR 6.43, 95%CI 1.03-40.06, p = 0.046  OR 0.31, 95%CI 0.09-0.99, p = 0.048 / not sig  OR 0.31, 95%CI 0.09-0.99, p = 0.048 / OR 13.49, 95%CI 1.76-103.07, p = 0.012  OR 0.21, 95%CI 0.06-0.71, p = 0.012 / not sig  OR 0.31, 95%CI 0.09-0.99, p = 0.048 / not sig  OR 12.5, 95%CI 3.33-50.00, p = 0.001 / OR 37.03, 95%CI 3.85-370.00, p = 0.002 |
| Brophy et al. [75] | IPAQ-SF | Low, moderate, high  MET-min/week | BASDAI  BASFI  Motivation to exercise (BREQ-2)  Previous motivation to exercise (BREQ-2)  Intrinsic (BREQ-2)  Identified (BREQ-2)  Amotivation (BREQ-2) | Descriptive stats  Path analysis | P = 0.0028 / non sig after controlling for motivation  Example of slope / coefficient, stratified by BASDAI and controlling for age: Mod BASDAI, Med PA on BASFI -15.4 (-24.4 to -6.5), p < .05  0.32, 95%CI 0.23-0.42  0.41, 95%CI 0.28-0.53  1320 (960 to 1680), p < 0.05  994 (651.5 to 1336), p < 0.05  -667 (-1242 to -92), p < 0.05 |
| Fabre et al. 76] | IPAQ-L | Level of PA  Meeting WHO PA  Guidelines | Being employed  Higher ASDAS | Univariate analysis  Multiple linear regression Univariate analysis  Multiple linear regression  Multivariate logistic regression | Not reported  β = −1998.4 ± 939.4, p = 0.03  β = 1012.1 ± 472.8, p = 0.03  Not sig  No variable sig |
| Fongen et al. [77] | IPAQ-L | HEPA  MET-min/week  MET-min/week | ASDAS-CRP  Season (summer) | Descriptive stats  ANCOVA  Wilcoxon signed rank test | p = 0.02  p = 0.02*  p ≤ 0.02 |
| Haglund et al. [78] | Customized (intensity, duration & No # days) | Meeting MVPA-rec  Meeting VI-PA-rec  Meeting MVPA-rec  Meeting VI-PA-rec  Meeting VI-PA-rec  Meeting MVPA-rec  Meeting VI-PA-rec  Meeting VI-PA-rec  Meeting MVPA-rec  Meeting VI-PA-rec | Age  Sex (female)  Previous smoker  BASDAI  BASFI  PsA subtype  EQ-5D | Multiple logistic regression | OR 1.02, 95%CI 1.01-1.03, p < 0.001  OR 0.99, 95%CI 0.98-1.00, p ≤0.01  OR ranging 1.63-1.78, p < 0.001  OR ranging from 0.68-0.73, p < 0.004  OR 0.92, 95%CI 0.87-0.98, p = 0.006  OR 0.94, 95%CI 0.90-0.98, p = 0.002  OR 0.87, 95%CI 0.83-0.91, p < 0.001  OR 0.72, 95%CI 0.56-0.92, p = 0.009  OR 2.76, 95%CI 1.54-4.95, p = 0.001  OR 6.42, 95%CI 3.12-13.2, p < 0.001 |
| Meesters et al. [79] | Customized (intensity, duration & No # days) | Meeting WHO rec | Depression (HADS) | Linear regression | B-est -0.97, 95%CI -1.32 to -0.62, p = 0.00  Cross-sectional (PA not the DV) |
| O’Dwyer et al. [80] | RT3 triaxial accelerometer | Health-enhancing PA bouts | V0_2MAX_ | Simple linear regression | R^2^ = 27.5 %, β = .524, p = .001 |
| Prince et al. [81] | Customized Interview | PA participation  Sport participation  Sport participation  Recreational PA  Overall PA  PA intensity  PA participation  PA intensity  Recreational PA  Sport participation  Recreational PA  Overall, PA  Overall PA intensity  Total hrs of PA | Having AS (before AS vs before anti-TNFa)  Taking Anti-TNFa (vs before)  Age of anti-TNFa start | McNemar’s test  Paired t—test  McNemar’s test  Paired t-test  Pearson correlation | Decline 25% - p = 0.002  Decline p > 0.001  Decline of 6.4 h/week, t = 7.204, p < 0.001  Decline of 2.8 h/week, t – 2.392, p = 0.02  Decline of 9.2 h/week, t = 5.853, p < 0.001  Decline of 36%, t = 5.571, p < 0.001  Increase of 14%, p = 0.039  Increase 33% p = 0.001  Increase, p = 0.004  Increase 1.8 hr/week, t = 3.411, p = 0.001  Increase 2.2 h/week, t = 4.289, p < 0.001  Increase 4.1 hr/week, t = 5.428, p < 0.001  r = -0.651, P = 0.001  r = –0.462, P = 0.001 |
| Van Genderen et al. [82] | Actigraph GT3X triaxial accelerometer | MVPA  Light PA  MVPA  MVPA | Diagnosis duration  BASDAI  BASFI  BMI | Multivariable regression* | B = -0.04, SE 0.02, p = 0.02, partial R = -0.21* (all ages)  B = 14.6, SE 7.2, p = 0.04, partial R = 0.25* (in ages ≥52 yrs)  B = -0.2, SE 0.09, p = 0.03, partial R = -0.19* (all ages)  B = -0.1, SE 0.04, p = 0.007, partial R = -0.24* (all ages) |
| Van Genderen et al. [83] | Tracmor triaxial accelerometer and Baecke Questionnaire | Baecke (total PA)  Total activity / day  Baecke (total PA)  Baecke (total PA)  Baecke (total PA)  Baecke (total PA) | Age  BASFI  Fatigue (MFI-Pf)  Fatigue (MFI-Mf)  Fatigue (MFI-Ra) | Spearman’s correlations | r = 0.51, p < 0.05  r = -0.47, p = 0.022  r = -0.39, p = 0.054 (counted as non-sig)  r = -0.54, p < 0.05  r = -0.48, p < 0.05  r = -0.54, p < 0.05 |

*Note*. Based on the review criteria and process, this table contains associations related to PA parameters that may not have been included within the associations table.
